# Genomics-Driven Discovery of Benzoxazole Alkaloids from the Marine-Derived *Micromonospora* sp. SCSIO 07395

**DOI:** 10.3390/molecules28020821

**Published:** 2023-01-13

**Authors:** Ziqian Cheng, Qingbo Zhang, Jing Peng, Xiaoyang Zhao, Liang Ma, Changsheng Zhang, Yiguang Zhu

**Affiliations:** 1 Key Laboratory of Tropical Marine Bio-Resources and Ecology, Guangdong Key Laboratory of Marine Materia Medica, South China Sea Institute of Oceanology, Innovation Academy for South China Sea Ecology and Environmental Engineering, Chinese Academy of Sciences, 164 West Xingang Road, Guangzhou 510301, China; 2 University of Chinese Academy of Sciences, 19 Yuquan Road, Beijing 100049, China; 3 Southern Marine Science and Engineering Guangdong Laboratory (Guangzhou), No.1119, Haibin Road, Nansha District, Guangzhou 511458, China; 4 Sanya Institute of Ocean Eco-Environmental Engineering, Yazhou Scientific Bay, Sanya 572000, China

**Keywords:** benzoxazole alkaloids, genome mining, heterologous expression, *Micromonospora* sp., marine natural products

## Abstract

Benzoxazole alkaloids exhibit a diverse array of structures and interesting biological activities. Herein we report the identification of a benzoxazole alkaloid-encoding biosynthetic gene cluster (*mich* BGC) in the marine-derived actinomycete *Micromonospora* sp. SCSIO 07395 and the heterologous expression of this BGC in *Streptomyces albus*. This approach led to the discovery of five new benzoxazole alkaloids microechmycin A–E (**1**–**5**), and a previously synthesized compound **6**. Their structures were elucidated by HRESIMS and 1D and 2D NMR data. Microechmycin A (1) showed moderate antibacterial activity against *Micrococcus luteus* SCSIO ML01 with the minimal inhibitory concentration (MIC) value of 8 μg mL^−1^.

## 1. Introduction

Heterocyclic compounds play an important role in drug discovery and development [1]. Benzoxazoles represent a family of heterocyclic compounds containing a benzene-fused oxazole ring core structure, which are frequently found in pharmaceutically active compounds [2], and natural products, for example caboxamycin [3], calcimycin [4], A33853 [5], nataxazole [6], and nocarbenzoxazoles [7] from actinomycetes, pseudopteroxazole [8] and nakijinol [9] from invertebrate, and salvianans [10,11] from plant. The unique structure and remarkable pharmaceutical potential of benzoxazoles have attracted significant attention for biosynthesis, including the construction of building blocks, the manner of backbone formation and subsequent heterocyclization and the mechanism of tailoring modifications (Figure S1) [12,13,14,15,16,17,18].

Heterologous expression of natural products biosynthetic gene clusters (BGCs) is of increasing interest in the discovery of novel bioactive compounds, which facilitates genome mining in the post-genomic era [19,20,21]. Our group previous research on genome mining by heterologous expression led to the isolation of twelve new polycyclic tetramate macrolactams from marine-derived *Streptomyces pactum* SCSIO 02999 and *Streptomyces* sp. SCSIO 40010 [22,23]. A rare actinomycete *Micromonospora* sp. SCSIO 07395, collected from the South China Sea at a depth of 96 m, was proven to be capable of production of a series of everninomicin derivatives with antimicrobial activity [24]. The genome sequence of this strain revealed the presence of more than twenty BGCs, suggesting a far greater potential to produce specialized metabolites than we have isolated. In this work, a dormant benzoxazole alkaloids encoding BGC from *Micromonospora* sp. SCSIO 07395 was activated through heterologous expression in *Streptomyces albus*, leading to five new compounds (**1**–**5**) and one previously synthesized compound (**6**).

## 2. Results and Discussion

**Figure 1:**
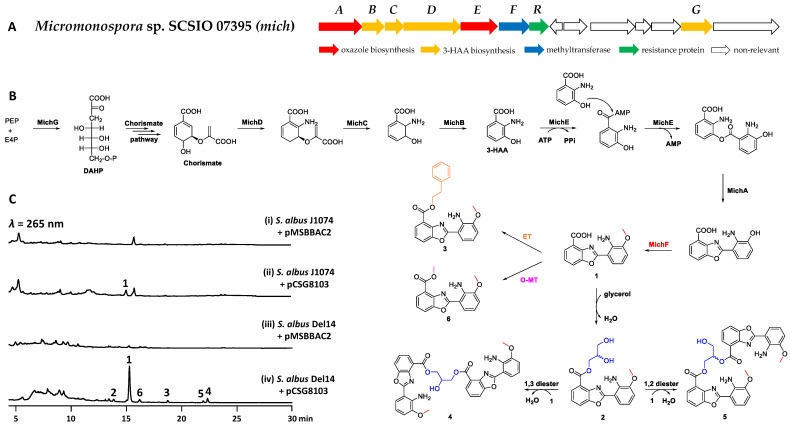
Biosynthesis of microechmycins. (**A**) The *mich* cluster identified from *Micromonospora* sp. SCSIO 07395. MichA, an amidohydrolase, MichB, a dehydrogenase, MichC, an isochorismatase, MichD, an anthranilate synthase, MichE, a coenzyme A ligase, MichF, a O-methyltransferase, MichR, a putative resistance-associated protein, MichG, a DAHP synthase. (**B**) Proposed biosynthetic pathway of microechmycins. ET: esterification, MT: methylation. (**C**) Product profiles from heterologous expression of *mich* BGC in *S. albus* J1074 and *S. albus* Del14.

In the course of genome mining to explore natural products from marine-derived actinomycetes, a BGC (*mich*, GenBank Accession NO. OP432093) encoded in *Micromonospora* sp. SCSIO 07395 predicated by antiSMASH [25] attracted our attention (Table S1 and Figure 1A). It contains five conserved genes (*michABCDE*) that are proposed to encode biosynthetic machinery to synthesize benzoxazole-containing compounds. The three enzymes MichBCD are homologous to BomOPQ that are involved in the biosynthesis of 3-hydroxyanthranilic acid (3-HAA) [12]; MichA and MichE (homologs of BomN and BomJ) are likely responsible for catalyzing dimerization of 3-HAA by ester formation and subsequent heterocyclization [17].

We attempted to investigate whether benzoxazole-containing compounds could be produced by the *mich* cluster by heterologous expression studies, since no such metabolites were detected in the fermentation of *Micromonospora* sp. SCSIO 07395. A pMSBBAC2-based [26] bacterial artificial chromosomal (BAC) genomic library of *Micromonospora* sp. SCSIO 07395 was constructed to screen positive clones (Table S2) covering the entire *mich* BGC by PCR with two primers pairs 8-F1/R1 and 8-F2/R2 (Table S3). Subsequently, the positive plasmid pCSG8103 was introduced into *Streptomyces albus* J1074 for heterologous expression. A new compound **1** was detected when compared to the control strain transformed with the empty BAC vector pMSBBAC2 (Figure 1C, trace i and ii), but the production of **1** was extremely low. *S. albus* Del14, the derivative of *S. albus* J1074, with deletion of 15 endogenous gene clusters has shown to be superior to the parental strain for heterologous production of natural products [27,28]. Then, the plasmid pCSG8103 was introduced into *S. albus* Del14. To our delight, in addition to the increased production of **1**, five additional compounds **2**–**6** were detected in *S. albus* Del14 harboring pCSG8103 (Figure 1C, trace iv). Therefore, a 15 L fermentation of *S. albus* Del14/pCSG8103 were performed for the isolation of compounds **1**–**6** (Figure 1B). As expected, compounds **1**–**6** were structurally established to be benzoxazole-containing compounds by extensive analysis of HRESIMS, 1D and 2D NMR data, named microechmycin A–F, respectively.

**Figure 2:**
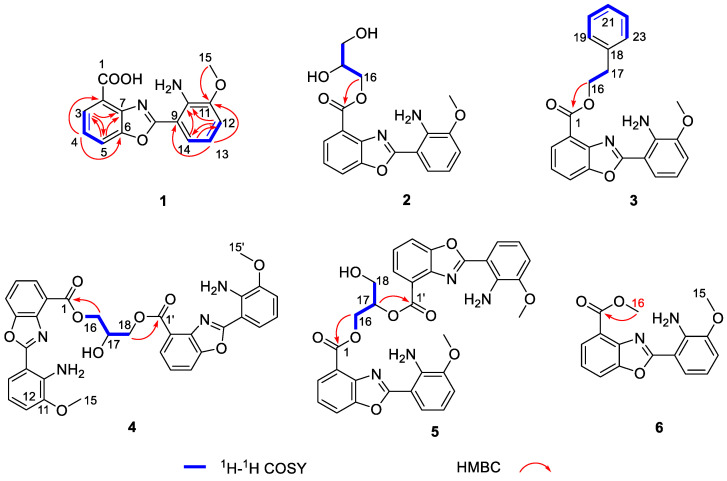
The Key ^1^H-^1^H COSY and HMBC correlations compounds **1**–**6**.

Microechmycin A (**1**) was isolated as yellow oil. The molecular formula of **1** was determined by HRESIMS *m*/*z*: [M − H]^−^, calcd for C_15_H_11_N_2_O_4_ 283.0724, found 283.0725, indicating 11 degrees of unsaturation (Figure S2). Analysis of the ^1^H, ^13^C, and heteronuclear single quantum coherence (HSQC) NMR data of **1** (Table 1 and Figure S2) showed the presence of signals for eight sp^2^ nonprotonated carbons, six sp^2^ methines, and one methoxy group (*δ*_C_/*δ*_H_ 55.7/2.89). The further inspection of the COSY spectrum of **1** revealed two ABC three-spin systems at *δ*_H_ 7.86 (H-3)/7.44 (H-4)/7.93 (H-5) and *δ*_H_ 7.02 (H-12)/6.69 (H-13)/7.57 (H-14) (Figure 2). Subsequently, two 1,2,3-substituted benzene moieties were deduced from HMBC correlations from H-3 to C-5/C-7, H-4 to C-2/C-6, H-5 to C-3/C-7, H-12 to C-10/C-14, H-13 to C-9/C-11, and H-14 to C-10/C-12 (Figure 2). Comparing the NMR data of **1** with the intermediate 2-(2-amino-3-hydroxyphenyl)benzoxazole-4-carboxylic acid in the biosynthetic pathway of A33853 [12,17] showed their high structural similarity. The only difference was the presence of signals for an additional methoxy group in **1**. The methoxy group was located at C-11 by the HMBC correlation from H_3_-15 to C-11. Finally, the structure of **1** was determined (Figure 2).

Microechmycin B (**2**) was isolated as yellow oil. The molecular formula of **2** was determined by HRESIMS *m*/*z*: [M + H]^+^, calcd for C_18_H_18_N_2_O_6_ 359.1239, found 359.1238 (Figure S3). Compound **2** displayed ^1^H and ^13^C NMR data that were similar to those of **1** (Table 1 and Figure S3). The major difference was the presence of NMR signals for an additional glycerol moiety in **2**. The presence of the glycerol moiety was supported by COSY correlations between H_2_-16 (*δ*_H_ 4.39, 4.27)/H-17 (*δ*_H_ 3.88) and H-17/H2-18 (*δ*_H_ 3.53). The glycerol moiety was linked to the carboxy group at C-1 in **1** to form an ester bond by HMBC correlations from H_2_-16 to C-1 (Figure 2). Finally, **2** was established as glycerol-ester derivative of **1**. The C-17 configuration of compound **2** failed to be established. Given that **2** is intermolecular esterification product between **1** and glycerol, it might be a racemic mixture, which was proved by chiral phase HPLC analysis with a mixture of two enantiomers with a ratio of 1:1 (**2a**:**2b**) (Figure S3A).

Microechmycin C (**3**) was isolated as yellow powder. The molecular formula of **3** was assigned to be C_23_H_20_N_2_O_4_ by HRESIMS (*m*/*z*: [M + H]^+^ at 389.1495, calcd for 389.1496) (Figure S4). Analysis of NMR data of **3** revealed that **3** was highly similar to **1**. The difference was that an additional phenylethyl group was present in **3** (Table 1 and Figure S4). The presence of the phenylethyl group was supported by COSY correlations between H_2_-16/H_2_-17, H-19/H-20, H-20/H-21, H-21/H-22 and H-22/H-23, and HMBC corre lations from H_2_-16 to C-18, H_2_-17 to C-18/C-19(C-23), and H-19(H-23) to C-16. Based on the HMBC correlation from H_2_-16 to C-1 (Figure 2), the phenylethyl group was connected to C-1 by an ester bond. Finally, the structure of **3** was elucidated (Figure 2).

Microechmycin D (**4**) was isolated as yellow oil. The molecular formula of **4** was determined by HRESIMS *m*/*z*: [M + H]^+^, calcd for C_33_H_29_N_4_O_9_ 625.1929, found 625.1931, indicating 22 degrees of unsaturation (Figure S5). Comprehensive analysis of the ^1^H, ^13^C and HSQC NMR data of **4** (Table 2 and Figure S5) revealed a pair of signals for eight sp^2^ nonprotonated carbons, six sp^2^ methines, one oxidized sp^3^ methylene group, one methoxy group, and the signal for an oxidized sp^3^ methine. Further analysis of 2D NMR data indicated that **4** was a dimeric compound connecting two monomers of **1** by glyceryl bridge through 1,3-diester bonds. This assignment was confirmed by the COSY correlations between H_2_-16(H-18)/H-17 and HMBC correlations from H_2_-16(H-18) to C-1(C-1’) (Figure 2). Therefore, the structure of compound **4** was established (Figure 2).

Microechmycin E (**5**) was isolated as yellow oil. The molecular formula of **5** was determined by HRESIMS *m*/*z*: [M + H]^+^, calcd for C_33_H_29_N_4_O_9_ 625.1929, found 625.1923 (Figure S6), which is similar to that of **4**. A detailed comparison of the NMR data of **5** and **4** (Table 2 and Figure S6) indicated that they were structurally highly similar and only differed from each other at the position forming the diester bonds. In **5**, the two monomers of **1** were connected by glyceryl bridge through 1,2-diester bonds, instead of the 1,3-diester bonds in **4**. The assignment was suggested by the COSY correlations between H_2_-16/H-17 and H-17/H_2_-18, and the HMBC correlations from H_2_-16 to C-1 and H-17 to C-1’ (Figure 2). Thus, the structure of compound **5** was determined. Although bearing a stereogenic center at C-17, **5** was shown to have an almost straight line in the electronic circular dichroism (ECD) spectrum (Figure S6A), thus indicating the presence of a racemate of **5**. A chiral-phase HPLC analysis of **5** further indicated that it was a mixture of two enantiomers with a ratio of 1:1 (**5a**:**5b**) (Figure S6A).

Microechmycin G (**6**) was isolated as yellow oil. The molecular formula of **6** was determined by HRESIMS *m*/*z*: [M + H]^+^, calcd for C_16_H_15_N_2_O_4_ 299.1026, found 299.1030 (Figure S7). Careful comparison of the NMR data demonstrated that the structures of **6** and **1** were highly similar (Table 1 and Figure S7). The only difference was the presence of an additional methoxy group (*δ*_C_/*δ*_H_ 52.4/4.03) in **6**, which was linked to the carboxy group at C-1 to form an ester bond. This assignment was supported by the HMBC correlations from H_3_-16 to C-1 (Figure 2). The NMR data of **6** was almost identical to those of a chemically synthesized compound [29], designated herein as Microechmycin G.

Based on the conserved biosynthetic mechanism for the formation of initial building block 3-HAA and heterocycle benzoxazole (Figure S1) [12,13,15,17], a putative pathway for microechmycins is outlined in Figure 1B. The 3-deoxy-D-arabino-heptulosonate 7-phosphate (DAHP) synthase MichG condenses phosphoenolpyruvate (PEP) and erythrose-4-phosphate (E4P) to yield DAHP, the first intermediate in the shikimate pathway leading to chorismate. 3-HAA is proposed to be derived from chorismate catalyzed by the anthranilate synthase MichD, isochorismatase MichC and the 2,3-dihydro-2,3-dihydroxybenzoate dehydrogenase MichB. Then, 3-HAA is condensed with a second 3-HAA by the ATP-dependent coenzyme A ligase MichE via an unstable ester intermediate followed by MichA-mediated benzoxazole formation. The methyltransferase MichF is responsible for the C-11 O-methylation to from compound **1**. The tailoring steps of microechmycins biosynthesis involve the attachment of glycerol and phenethyl alcohol moiety to the benzoxazole core structure of **1** via esterification at the C-1 carboxyl leading to compounds **2**, **3** or O-methylation to form **6**. The second esterification between **3** and **1** to produce **4** and **5** through 1,3 diester or 1,2 diester. However, no genes encoding enzymes for esterification were identified in the *mich* BGC, suggested the esterification could be catalyzed by endogenous enzymes from the heterologous host *S. albus* Del14.

Compounds **1**–**6** were evaluated for their antibacterial activities against two Gram positive bacteria *Staphylococcus aureus* ATCC 29213, *Micrococcus luteus* SCSIO ML01 and four Gram negative bacteria *Escherichia coli* ATCC 25922, *Vibrio alginolyticus* 13214, *Acinetobacter baumannii* ATCC 19606 and *Klebsiella. pneumoniae* ATCC 13883 by the broth micro dilution method [30]. Compound **1** showed moderate antibacterial activity only against *M. luteus* SCSIO ML01 with minimal inhibitory concentration (MIC) of 8 μg mL^−1^. However, compounds **2**–**6** did not exhibit any antibacterial activity for the tested strains.

## 3. Materials and Methods

### 3.1. Bacterial Strains and Reagents

Bacterial strains and plasmids used and constructed in this study are listed in Table S2. The strain *Streptomyces albus* Del14 [27] was obtained from Prof. Andriy Luzhetskyy in University of Saarland, Germany and Prof. Hailong Wang in Shandong University, China. The plasmid pMSBBAC2 [26] was obtained from Prof. Jing He in Huazhong Agricultural University, China. Chemicals and molecular biological reagents were purchased from standard commercial sources and used according to the manufacturer’s recommendations.

### 3.2. General Experimental Procedures

Optical rotations were measured using a 341 Polarimeter (Perkin-Elmer, Inc. Norwalk, CT, USA). CD spectra were measured on a Chirascan circular dichroism spectrometer (Applied Photophysics, Ltd. Surrey, UK). UV spectra were measured on a U-2600 spectrophotometer (Shimadzu, Tokyo, Japan). 1D and 2D NMR spectra were recorded on a Bruker AV-700 MHz NMR spectrometer (Bruker Biospin GmbH, Rheinstetten, Germany) with tetramethylsilane (TMS) as the internal standard. Mass spectrometric data were obtained on a quadrupole-time-of-flight mass spectrometry (Bruker Maxis 4G, Rheinstetten, Germany) for high-resolution electrospray ionization mass spectrometric (HRESIMS). Column chromatography was performed using silica gel (100–200 mesh, Jiangyou Silica gel development, Inc., Yantai, China), Sephadex LH-20 (Amersham pharmacia Biotech AB, Staffanstorp, Sweden).

### 3.3. General DNA Manipulation Techniques

All DNA manipulations in this study were performed according to manufacturers’ protocols. PCR was performed according to recommended protocol using *EasyTaq* (TransGen Biotech, Beijing, China). DNA sequencing was performed at Shanghai Majorbio Bio-Pharm Technology Co., Ltd. (Shanghai, China). Primers used in this study (Table S3) were synthesized at IGE biotech (Guangzhou, China).

### 3.4. Sequence Analysis

The BGCs were determined using antismash (https://antismash.secondarymetabolites.org) and *orfs* were determined using 2ndfind program (http://biosyn.nih.go.jp/2ndfind/) accessed on 27 March 2021. Protein sequences were compared with BLAST programs (http://blast.ncbi.nlm.nih.gov/Blast.cgi) accessed on 1 April 2021.

### 3.5. Heterologous Expression of Mich BGC

The BAC library of *Micromonospora* sp. SCSIO 07395 was constructed using the BAC vector pMSBBAC2 [26] by Eight Star Bio-tech company (Eight Star Bio-tech Co., Ltd. Wuhan China). The positive clone pCSG8103 containing the *mich* BGC was screened out by PCR with primers 8-F1/R1 and 8-F2/R2 (Table S3). *S. albus* Del14 [27] and *S. albus* J1074 [31] (Table S2) were used as the hosts for heterologous expression. *E. coil* ET12567/pUB307 [32] was used as the helper strains for the triparental mating. The heterologous hosts were grown at 28 °C in MS medium (soybean powder 20 g·L^−1^, mannitol 20 g·L^−1^, agar powder 20 g·L^−1^, pH 7.2) for growth and sporulation. The *E. coli* strains were grown in Luria-Bertani medium at 37 °C. The plasmid pCSG8103 was introduced into the host strain *S. albus* J1074 and *S. albus* Del14 by conjugation with the help of *E. coli* ET12567/pUB307. Three positive clones were randomly selected for small scale fermentation and subjected to metabolite analyses by HPLC.

### 3.6. Fermentation and Metabolite Analyses by HPLC

The fermentation of the recombinant strains was carried out using 4^#^ (fish peptone 8 g·L^−1^, soluble starch 15 g·L^−1^, cornmeal 8 g·L^−1^, tryptone 5 g·L^−1^, glycerol 5 g·L^−1^, CaCO_3_ 2 g·L^−1^, KBr 0.2 g·L^−1^, artificial sea salt 30 g·L^−1^, PH 7.2-7.4) medium in 250 mL flask by shaking at 200 rpm and 28 °C for 4 days. A 5 mL fermentation broth was extracted with 5 mL butanone, and the extract was then concentrated under vacuum. The residues were dissolved into 100 μL of methanol. For analyzing the metabolites of recombinant strains, the HPLC was used by Agilent 1260 infinity series instrument (Agilent Technologies Inc. Santa Clara, USA) and a reversed phase column (Phenomenex kinetex, C18, 5 μm, 250 mm × 4.6 mm) with UV detection at 265 nm under the following program: solvent system (solvent A, 10% CH_3_CN in water supplemented with 0.08% formic acid; solvent B, 90% CH_3_CN in water), 5% B to 100% B (0–20 min), 100% B (21–25 min), 100% B to 5%B (25–26 min), 5% B (26–30 min), flow rate at 1 mL min^−1^.

### 3.7. Large-Scale Fermentation and Isolation of Compounds from Heterologous Expression Strain

The 15 L of culture broth of *S. albus* Del14/pCSG8103 were pooled and centrifuged at 3900 rpm for 15 min at 25 °C. The mycelia were extracted three times, each with 2 L acetone. The acetone extracts were concentrated under reduced pressure to afford an aqueous residue, which was extracted three times with equal volume butanone. The supernatant was extracted three times with equal volume butanone. The butanone extracts were combined and concentrated under reduced pressure to afford crude extract (9.86 g). Subsequently, the crude extract was subjected to a (100–200 mesh) silica gel column eluting with a gradient of petroleum ether/acetone mixture from 50:1, 20:1, 10:1, 5:1 and 1:1 yielded 27 fractions (Fr.1-Fr.27). The fractions Fr.18 and Fr.19 were combined further separated by silica gel column (100–200 mesh) and eluted with CHCl_3_/CH_3_OH (50:1, *v*/*v*) to obtain 7 sub-fractions (Fr.A1 to Fr.A7). The fractions Fr.A2 and Fr.A3 were combined (0.83 g) and further separated by Sephadex LH-20 and eluted with petroleum ether/CHCl_3_/CH_3_OH (5:5:1, *v*/*v*) to yield 16 sub-fractions (Fr.B1 to Fr.B16). Then, Fr.B4 to Fr.B9 were combined and further separated by semi-preparative HPLC to yield **1** (99.2 mg), **4** (4.1 mg) and **5** (4.0 mg). Fr.7 was separated by Sephadex LH-20 and eluted with petroleum ether/CHCl_3_/CH_3_OH (5:5:1, *v*/*v*) to yield 15 subfractions (Fr.7.1 to Fr.7.15). Fr.7.8 and Fr.7.9 were combined and separated by semi-preparative HPLC to yield compound **6** (3.8 mg) and **3** (3.5 mg). Fr.9 was separated by Sephadex LH-20, eluted with petroleum ether/CHCl_3_/CH_3_OH (5:5:1, *v*/*v*) to yield 13 subfractions (Fr.9.1 to Fr.9.13). Fr9.7 to Fr.9.10 were combined and separated by semi-preparative HPLC to yield compound **2** (5.2 mg).

Microechmycin A (**1**): C_15_H_11_N_2_O_4_; yellow oil; UV (CH_3_CN) λ_max_ (log *ε*): 202.60 (3.97), 234.80 (3.95), 272 (3.76), 285 (3.74), 309.40 (3.81), 372 (3.75) nm; Data of ^1^H (700 MHz, DMSO-*d*6) and ^13^C NMR (175 MHz, DMSO-*d*6), see Table 1; HR-ESI-MS: *m*/*z* 283.0725 [M − H]^−^ (calcd. for C_15_H_11_NO_4_, 283.0724).

Microechmycin B (**2**): C_18_H_18_N_2_O_6_; yellow oil; ${\left[\alpha \right]}_{D}^{25}$-2.00 (c 1, MeOH); UV (CH_3_CN) λ_max_ (log *ε*): 202.60 (3.96), 234.60 (3.71), 271.80 (3.36), 284.80 (3.25), 320 (3.41), 378.20 (3.26) nm; Data of ^1^H (700 MHz, DMSO-*d*6) and ^13^C NMR (175 MHz, DMSO-*d*6), see Table 1; HR-ESI-MS: *m*/*z* 359.1238 [M + H]^+^ (calcd. for C_18_H_18_N_2_O_6_, 359.1239).

Microechmycin C (**3**): C_23_H_20_N_2_O_4_; yellow powder; UV (CH_3_CN) λ_max_ (log *ε*): 204 (4.12), 271.80 (3.34), 284 (3.25), 310 (3.33), 371.20 (3.18); Data of ^1^H (700 MHz, DMSO-*d*6) and ^13^C NMR (175 MHz, DMSO-*d*6), see Table 1; HR-ESI-MS: *m*/*z* 389.1495 [M + H]^+^ (calcd. for C_23_H_20_N_2_O_4_, 389.1496).

Microechmycin D (**4**): C_33_H_29_N_4_O_9_; yellow oil; UV (CH_3_CN) λ_max_ (log *ε*): 202.40 (4.00), 234.60 (4.05), 272 (4.00), 284.80 (4.03), 320.40 (4.19), 378.20(4.28) nm; Data of ^1^H (700 MHz, CDCl_3_) and ^13^C NMR (175 MHz, CDCl_3_), see Table 2; HR-ESI-MS: *m*/*z* 625.1931 [M + H]^+^ (calcd. for C_33_H_29_N_4_O_9_, 625.1929).

Microechmycin E (**5**): C_33_H_29_N_4_O_9_; yellow oil; [*α*]_25_ + 7.50 (c 1, MeOH); UV (CH_3_CN) λ_max_ (log *ε*): 221.60 (3.06), 234.80 (3.19), 271 (3.16), 286 (3.01), 311.80 (3.10), 320.80 (3.48) nm, 375.60 (3.44); Data of ^1^H (700 MHz, CDCl_3_) and ^13^C NMR (175 MHz, CDCl_3_), see Table 2; HR-ESI-MS: *m*/*z* 625.1923 [M + H]^+^ (calcd. for C_33_H_29_N_4_O_9_, 625.1929).

Microechmycin F (**6**): C_16_H_15_N_2_O_4_; yellow oil; UV (CH_3_CN) λ_max_ (log *ε*): 235.40 (2.87), 270.20 (2.99), 284.80 (2.94), 309 (2.77), 319.40 (2.86), 375 (3.22) nm; Data of ^1^H (700 MHz, CDCl_3_) and ^13^C NMR (175 MHz, CDCl_3_), see Table 1; HR-ESI-MS: *m*/*z* 299.1030 [M + H]^+^ (calcd. for C_16_H_15_N_2_O_4_, 299.1026).

### 3.8. Chiral HPLC Analysis

Chiral HPLC analysis of compound **2** was conducted by using a chiral column (Lux Cellulose-3, 5 μm, 250 × 4.6 mm, phenomenex) with UV detection at 265 nm under the following program: solvent system (solvent A, 10% CH_3_CN in water; solvent B, 90% CH_3_CN in water); 38% B (0–35 min), flow rate at 1 mL min^−1^. Chiral HPLC analysis of compound **5** was conducted by using a chiral column (Lux Cellulose-4, 5 μm, 250 × 4.6 mm, phenomenex) with UV detection at 265 nm under the following program: solvent system (solvent A, 10% CH_3_CN in water; solvent B, 90% CH_3_CN in water); 5% B to 100% B (0–20 min), 100% B (21−25 min), 100% B to 5%B (25−26 min), 5% B (26–30 min), flow rate at 1 mL min^−1^.

### 3.9. Bioactivity Assays

Antibacterial activity of compounds **1**–**6** was measured against two Gram positive bacteria *S. aureus* ATCC 29213, *M. luteus* SCSIO ML01 and four Gram negative bacteria *E. coli* ATCC 25922, *V. alginolyticus* 13214, *A. baumannii* ATCC 19606 and *K. pneumoniae* ATCC 13883 by the broth micro dilution method [30]. Ciprofloxacin was used as positive control.

## 4. Conclusions

In summary, we have expressed the *mich* gene cluster from *Micromonospora* sp. SCSIO 07395 in the heterologous *S. albus* host and identified the benzoxazole-containing microechmycins (**1**–**6**) as the products of the pathway. Microechmycin D (**4**) and E (**5**) can be synthesized through the actions of esterification between glycerol and two molecules of **1**. This glycerol esterification has been observed in acautalides C discovery from *Acaulium* sp H-JQSF [33]. The discovery of microechmycins adds to the inventory of secondary metabolites that can be biosynthesized by *Micromonospora* sp. SCSIO 07395, and highlights the potential of searching for novel natural products from marine rare actinomycetes by heterologous expression.

## Figures and Tables

**Table 1:** The NMR data of compounds **1**–**3** and **6** (*J* in Hz).

No.	1 ^a^		2 ^a^		3 ^a^		6 ^b^	
	*δ*_C_	*δ*_H_	*δ*_C_	*δ*_H_	*δ*_C_	*δ*_H_	*δ*_C_	*δ*_H_
1	166.9, C		164.5, C		164.3, C		166.1, C	
2	124.2, C		120.7, C		120.3, C		121.4, C	
3	126.3, CH	7.86, d (7.7)	126.6, CH	7.96, dd (7.7, 0.7)	126.3, CH	7.88, d (7.7)	126.8, CH	8.00, d (7.7)
4	124.2, CH	7.44, dd (7.7, 7.7)	124.4, CH	7.51, dd (7.7, 7.7)	124.5, CH	7.49, dd (7.7, 7.7)	123.9, CH	7.37, dd (7.7, 7.7)
5	113.7, CH	7.93, d (7.7)	115.1, CH	8.03, dd (7.7, 0.7)	114.9, CH	8.03, d (7.7)	114.6, CH	7.74, d (7.7)
6	149.2, C		149.2, C		149.4, C		150.1, C	
7	140.5, C		140.8, C		140.6, C		141.8, C	
8	163.7, C		164.2, C		164.3, C		165.1, C	
9	105.6, C		113.3, C		105.1, C		107.1, C	
10	139.7, C		139.5, C		139.9, C		140.0, C	
11	146.8, C		147.0, C		146.8, C		147.2, C	
12	112.5, CH	7.02, dd (7.7, 0.7)	112.4, CH	7.03, d (7.7)	112.6, CH	7.03, d (7.7)	112.1, CH	6.88, d (7.7)
13	114.9, CH	6.69, dd (7.7, 8.4)	114.9, CH	6.69, dd (7.7, 8.4)	115.1, CH	6.69, dd (7.7, 7.4)	115.4, CH	6.72, dd (7.7, 7.7)
14	119.5, CH	7.57, dd (8.4, 1.4)	119.4, CH	7.57, dd (8.4, 1.4)	119.4, CH	7.57, d (7.7)	120.3, CH	7.68, (7.7)
15	55.7, CH_3_	3.87, s	55.7, CH_3_	3.90, s	55.7, CH_3_	3.88, s	55.9, CH_3_	3.94, s
16			66.0, CH_2_	4.39, dd (11.2, 4.2); 4.27, dd (11.2, 5.6)	65.3, CH_2_	4.56, dd (14.0, 7.0)	52.4, CH_3_	4.03, s
17			69.2, CH	3.88, m	34.4, CH_2_	3.14, m		
18			62.8, CH_2_	3.53, m	138.0, C			
19					129.0, CH	7.38, d (7.7)		
20					128.4, CH	7.31, dd (7.7, 7.7)		
21					126.4, CH	7.22, dd (7.7, 7.7)		
22					128.4, CH	7.31, dd (7.7, 7.7)		
23					129.0, CH	7.38, d (7.7)		

^a^ 700 MHz for ^1^H, 175 MHz for ^13^C, DMSO-*d_6_*, ^b^ 700 MHz for ^1^H, 175 MHz for ^13^C, CDCl_3_, TMS as an internal standard.

**Table 2:** The NMR data of compounds **4** and **5** (*J* in Hz).

No.	4		5	
	*δ*_C_	*δ*_H_	*δ*_C_	*δ*_H_
1	165.8, C		165.8, C	
2	120.7, C		120.5, C	
3	127.0, CH	7.97, d (7.7)	127.1, CH	7.99, d (7.7)
4	123.9, CH	7.33, dd (7.7, 7.7)	123.8, CH	7.33, dd (7.7, 7.7)
5	114.8, CH	7.71, d (7.7)	114.7, CH	7.70, d (7.7)
6	149.9, C		150.0, C	
7	141.7, C		141.8, C	
8	163.2, C		164.8, C	
9	106.9, C		106.9, C	
10	139.9, C		139.8, C	
11	147.2, C		147.3, C	
12	112.1, CH	6.71, d (7.7)	112.3, CH	6.83, d (7.7)
13	116.0, CH	6.61, dd (7.7, 7.7)	115.6, CH	6.68, dd (7.7, 7.7)
14	120.3, CH	7.52, (7.7)	120.2, CH	7.60, (7.7)
15	55.8, CH_3_	3.71, s	55.8, CH_3_	3.86, s
16	66.3, CH_2_	4.72, m	63.4, CH_2_	4.91, m; 4.83, m
17	68.5 CH	4.59, m	73.5 CH	5.63, m
18	66.3, CH_2_	4.72, m	61.9 CH_2_	4.14, m
1’	165.8, C		165.8, C	
2’	120.7, C		120.5, C	
3’	127.0, CH	7.97, d (7.7)	127.1, CH	7.99, d (7.7)
4’	123.9, CH	7.33, dd (7.7, 7.7)	123.8, CH	7.33, dd (7.7, 7.7)
5’	114.8, CH	7.71, d (7.7)	114.7, CH	7.70, d (7.7)
6’	149.9, C		150.0, C	
7’	141.7, C		141.8, C	
8’	163.2, C		164.8, C	
9’	106.9, C		106.9, C	
10’	149.9, C		139.8, C	
11’	147.2, C		147.3, C	
12’	112.1, CH	6.71, d (7.7)	112.3, CH	6.83, d (7.7)
13’	116.0, CH	6.61, dd (7.7, 7.7)	115.6, CH	6.68, dd (7.7, 7.7)
14’	120.3, CH	7.52, (7.7)	120.2, CH	7.60, (7.7)
15’	55.8, CH_3_	3.71, s	55.8, CH_3_	3.86, s

## Data Availability

Not applicable.

## Supplementary Materials

The following supporting information can be downloaded at: https://www.mdpi.com/article/10.3390/molecules28020821/s1, Table S1. Deduced functions of individual *orf*s in the *mich* gene cluster. Table S2. Strains and plasmids were used and generated in this study. Table S3. Primers were used in this study. Table S4. The antibacterial activities of compound **1**–**6**. Figure S1. The representative biosynthetic pathway of benzoxazoles. Figure S2. The spectroscopic data of **1**. Figure S3. The spectroscopic data of **2**. Figure S4. The spectroscopic data of **3**. Figure S5. The spectroscopic data of **4**. Figure S6. The spectroscopic data of **5**. Figure S7. The spectroscopic data of **6**. Refs [26,27,31,32] are cited in the Supplementary Materials.

## Sample Availability

Samples of the compounds are not available.
