# Supplementary material for: Genomics-Driven Discovery of Benzoxazole Alkaloids from the Marine-Derived Micromonospora sp. SCSIO 07395
Source: Molecules. 2023 Jan 13;28(2):821. doi: 10.3390/molecules28020821 (PMC9864271; doi:10.3390/molecules28020821)
Supplement: Supplementary file 1 [file molecules-28-00821-s001.zip › molecules-2130366-SI.pdf]

Supporting Information

Genomics-Driven Discovery of Benzoxazole Alkaloids from the Marine-Derived *Micromonospora* sp. SCSIO 07395

Ziqian Cheng [1,2,†], Qingbo Zhang [1,2,3,4,†], Jing Peng [1,2,†], Xiaoyang Zhao [1,2], Liang Ma [1,3], Changsheng Zhang [1,2,3,4,*] and Yiguang Zhu [1,2,3,4,*]

1 Key Laboratory of Tropical Marine Bio-Resources and Ecology, Guangdong Key Laboratory of Marine
Materia Medica, South China Sea Institute of Oceanology, Innovation Academy for South China Sea
Ecology and Environmental Engineering, Chinese Academy of Sciences, 164 West Xingang Road,
Guangzhou 510301, China

2 University of Chinese Academy of Sciences, 19 Yuquan Road, Beijing 100049, China

3 Southern Marine Science and Engineering Guangdong Laboratory (Guangzhou), No.1119, Haibin Road,
Nansha District, Guangzhou 511458, China

4 Sanya Institute of Ocean Eco-Environmental Engineering, Yazhou Scientific Bay, Sanya 572000, China

* Correspondence: czhang@scsio.ac.cn (C.Z.); ygzhu@scsio.ac.cn (Y.Z.); Tel.: +86-20-8902-3038 (C.Z. and Y.Z.)

† These authors contribute equally to this work.

Table of Contents

Table S1. Deduced functions of individual *orfs* in the *mich* gene cluster 3
Table S2. Strains and plasmids used and generated in this study 4
Table S3. Primers used in this study 5
Table S4. The antibacterial activities of compound **1-6** (MIC, μg·mL^{-1}) 6
Figure S1 The representative biosynthetic pathway of benzoxazole 7
Figure S2. The spectroscopic data of **1** 8
Figure S3. The spectroscopic data of **2** 15
Figure S4. The spectroscopic data of **3** 23
Figure S5. The spectroscopic data of **4** 30
Figure S6. The spectroscopic data of **5** 37
Figure S7. The spectroscopic data of **6** 45
References 52

Table S1. Deduced functions of individual *orfs* in the *mich* gene cluster

Gene	Size (aa)	Proposed protein function	Orthologue in A33853 cluster (% deduced protein identity)	Orthologue in caboxamycin cluster (% deduced protein identity)	Orthologue in nataxazole cluster (% deduced protein identity)
orf-2	381	Probable acyl-CoA dehydrogenase			
orf-1	188	Siroheme synthase			
michA	494	amidohydrolase	*bomN* (60%)	*cbxE* (58%)	*natAM* (60%)
michB	254	2,3-dihydro-2,3-dihydroxybenzoate dehydrogenase	*bomO* (61%)	*cbxG* (63%)	*natDB* (61%)
michC	215	Isochorismatase	*bomP* (55%)	*cbxH* (63%)	*natIS* (59%)
michD	659	Anthranilate synthase	*bomQ* (64%)	*cbxI* (58%)	*natAN* (57%)
michE	427	coenzyme A ligase	*bomJ* (64%)	*cbxC* (57%)	*NatL2* (63%)
michF	349	O-methyltransferase			
michR	224	YdeI/OmpD-associated family protein			
orf1	140	50S ribosomal protein			
orf2	275	Arylamine N-acetyltransferase			
orf3	522	aldehyde dehydrogenase			
orf4	177	SRPBCC family protein			
orf5	341	ketoacyl-ACP synthase III family protein			
michG	362	DAHP synthase			
orf6	776	non-ribosomal peptide synthetetase			

Table S2. Strains and plasmids used and generated in this study

**Strains/Plasmids**	**Characteristics**	**Sources**
Escherichia coli		
DH10B	Host strain for BAC library construction	Giboco BRL
ET12567/pUB307	Host strain for conjugation	[41]
**Actinomycetes**		
Micromonospora sp. SCSIO 07395	Wild type	This study
S. albus J1074	Host strain for heterologous expression	[32]
S. albus Del14	Host strain for heterologous expression	[13]
S. albus J1074/pMSBBAC2	*S. albus* J1074 containing plasmid pMSBBAC2	This study
S. albus J1074/pCSG8103	*S. albus* J1074 containing plasmid pCSG8103	This study
S. albus Del14/pMSBBAC2	*S. albus* Del14 containing plasmid pMSBBAC2	This study
S. albus Del14/pCSG8103	*S. albus* Del14 containing plasmid pCSG8103	This study
**Plasmids**		
pMSBBAC2	Apr^r, the vector for BAC library construction	[24]
pCSG8103	Apr^r, BAC plasmid containing entire microechmycin (*mich*) BGC	This study

Table S3. Primers used in this study

8-F1	CGTCGTCCGGCCACCCATTGATA
8-R1	CCCCCGGCCGCATTACTGTGAA
8-F2	GCCACCGCAGTCCACCGTCATTTC
8-R2	CCAGGGCCCGGATCAGGTTGTTCA

Table S4. The antibacterial activities of compound **1-6** (MIC, μg·mL^{-1})

Compounds	*S. aureus* ATCC 29213	*M. luteus* SCSIO ML01	*E. coli* ATCC 25922	*V. alginolyticus* 13214	*A.baumannii* ATCC 19606	*K. pneumoniae* ATCC 13883
**1**	>64	8	>64	>64	>64	>64
**2**	>64	>64	>64	>64	>64	>64
**3**	>64	>64	64	>64	>64	>64
**4**	>64	>64	>64	>64	>64	>64
**5**	>64	>64	>64	>64	>64	>64
**6**	>64	>64	>64	>64	>64	>64
Ciprofloxacin	0.25	1	0.0125	0.03	0.125	0.03

Note： Ciprofloxacin is a positive control.

Figure S1 The representative biosynthetic pathway of benzoxazole

(A) The biosynthesis of A33853

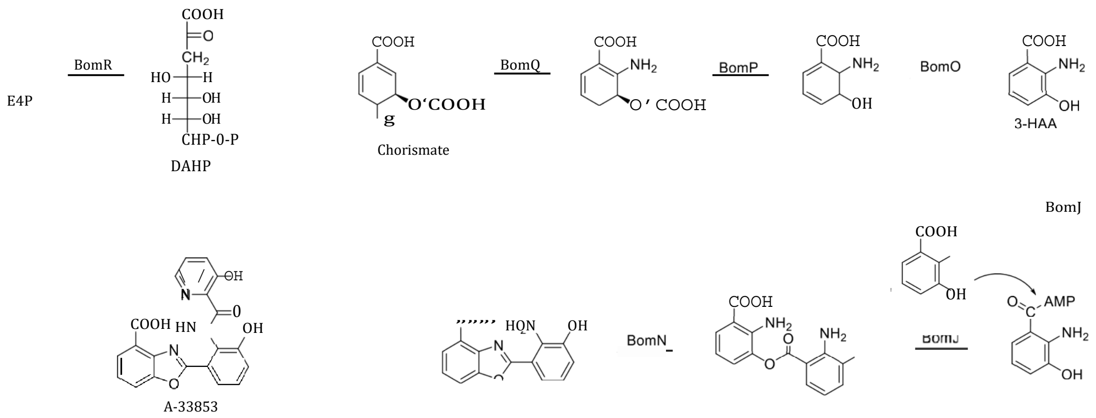

(B) The biosynthesis of nataxazole

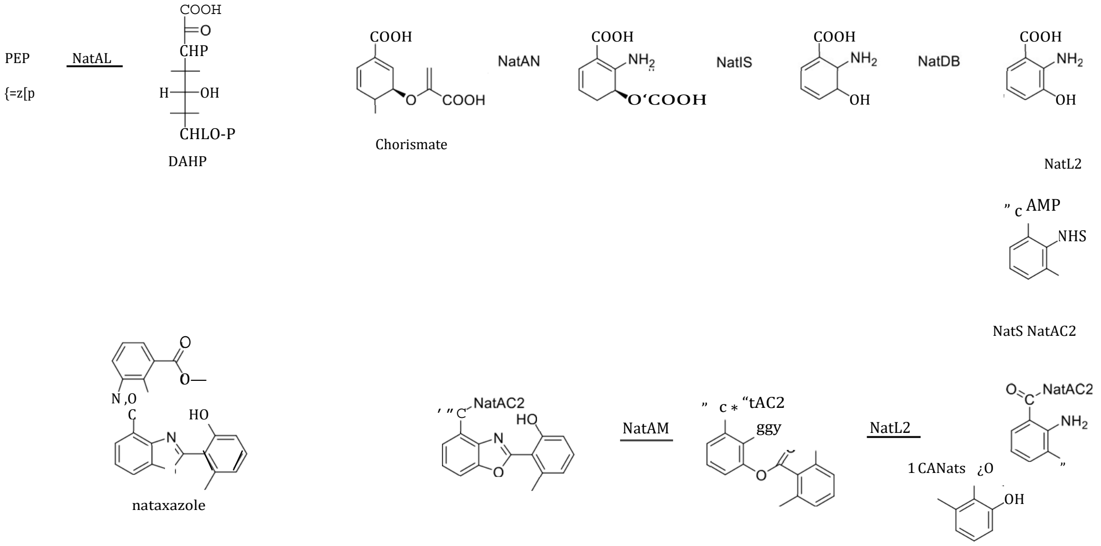

(C) The biosynthesis of caboxamycin

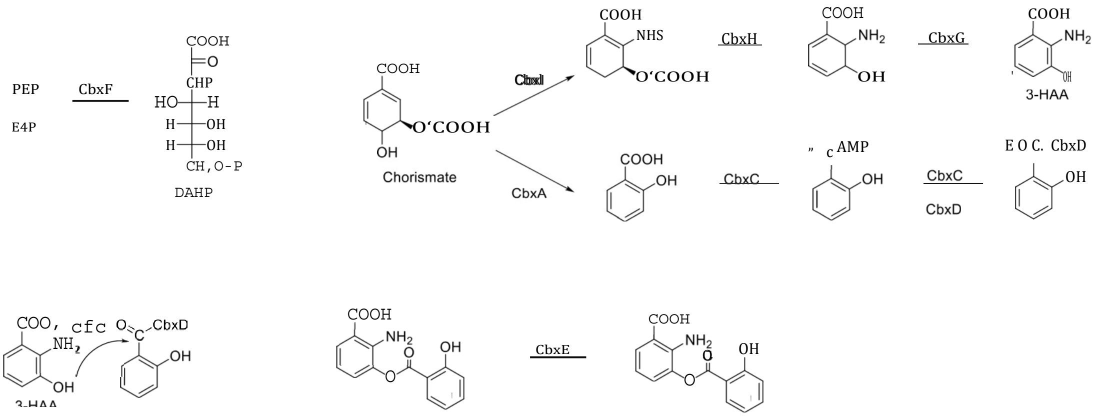

Figure S2. The spectroscopic data of **1**

(A) The HRESIMS (a) UV (b) spectrum of **1**

(a)

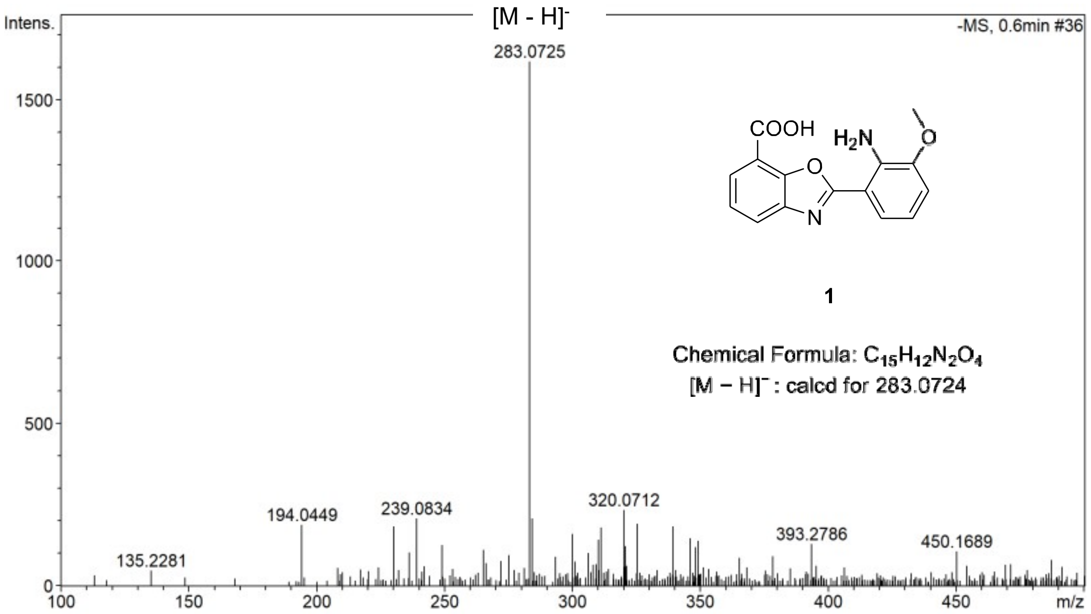

(b)

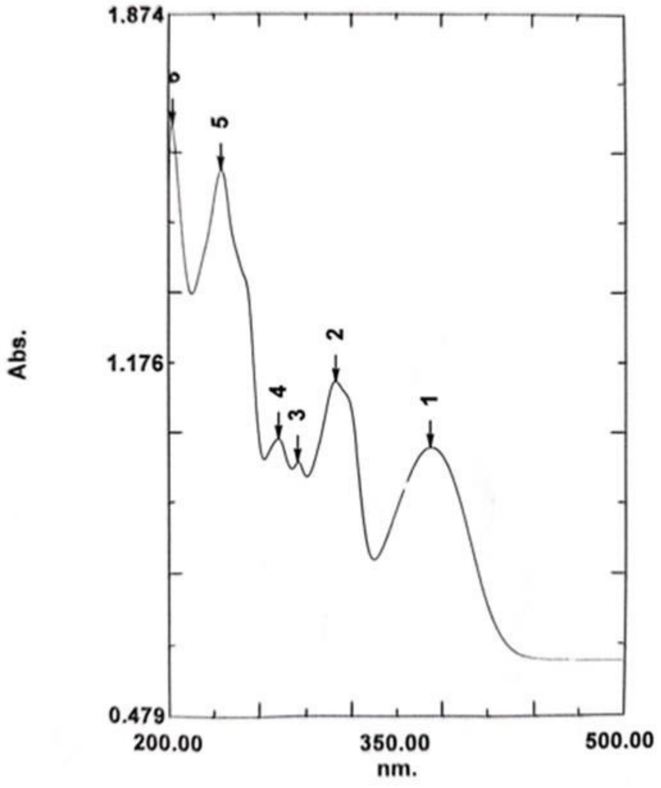

Figure S2. The spectroscopic data of **1**

(B) The ^{1}H-NMR spectrum of **1** (700 MHz for ^{1}H NMR in DMSO-d_6)

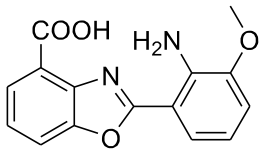

1

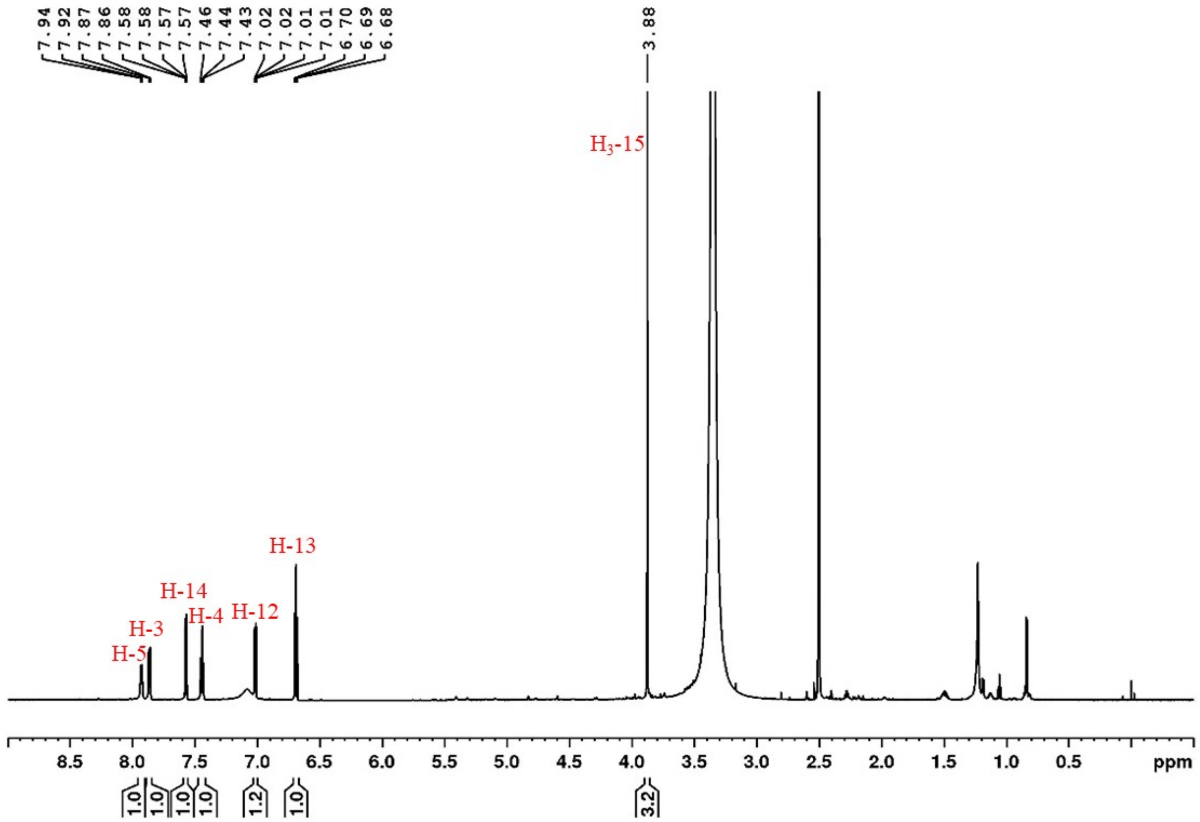

Figure S2. The spectroscopic data of **1**

(C) The^{13}C-NMR spectrum of compound **1** in DMSO-d_6 (175 MHz)

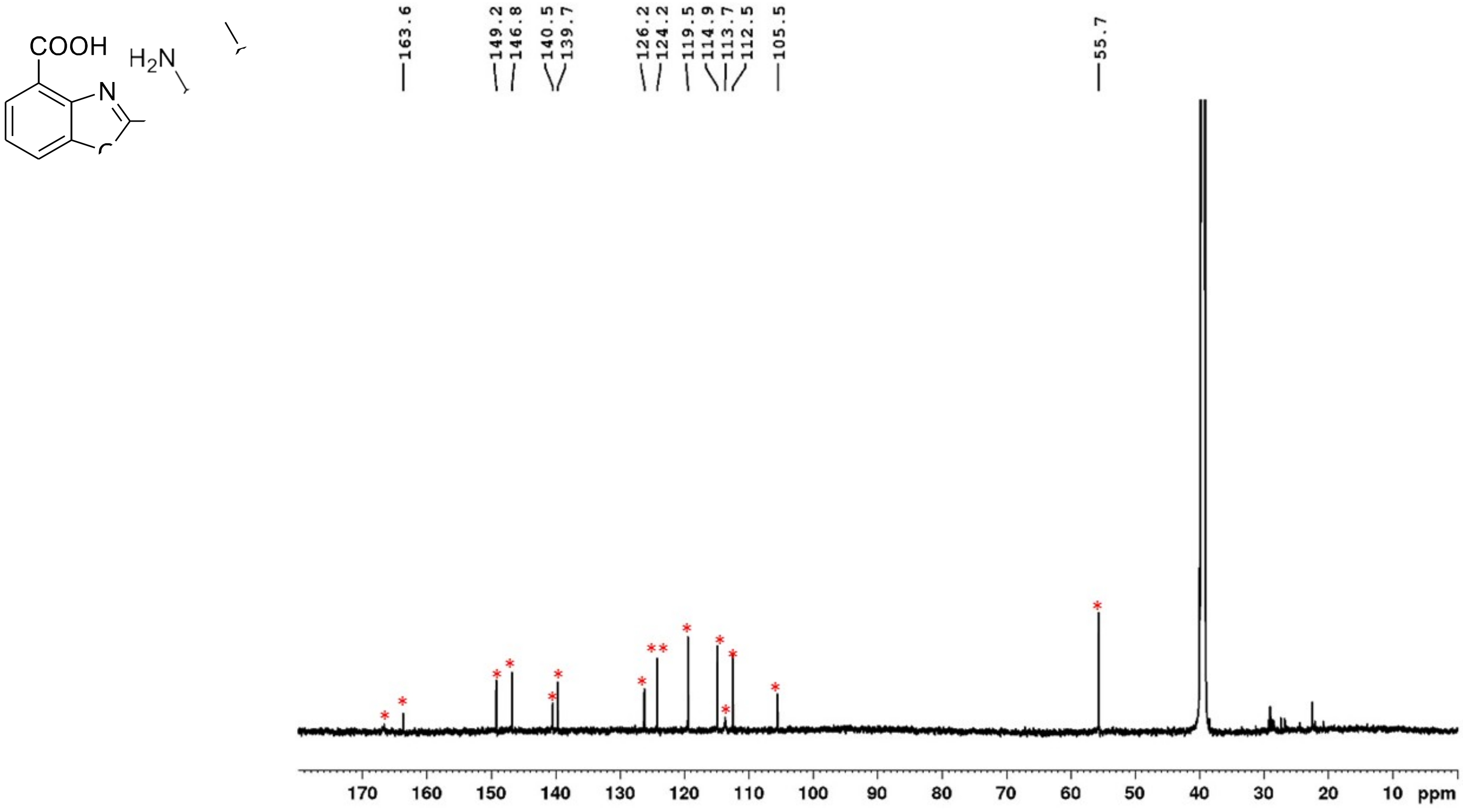

Figure S2. The spectroscopic data of **1**

(D) The DEPT135 spectrum of **1** (175MHz, DMSO-d_6)

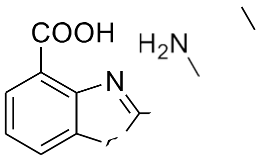

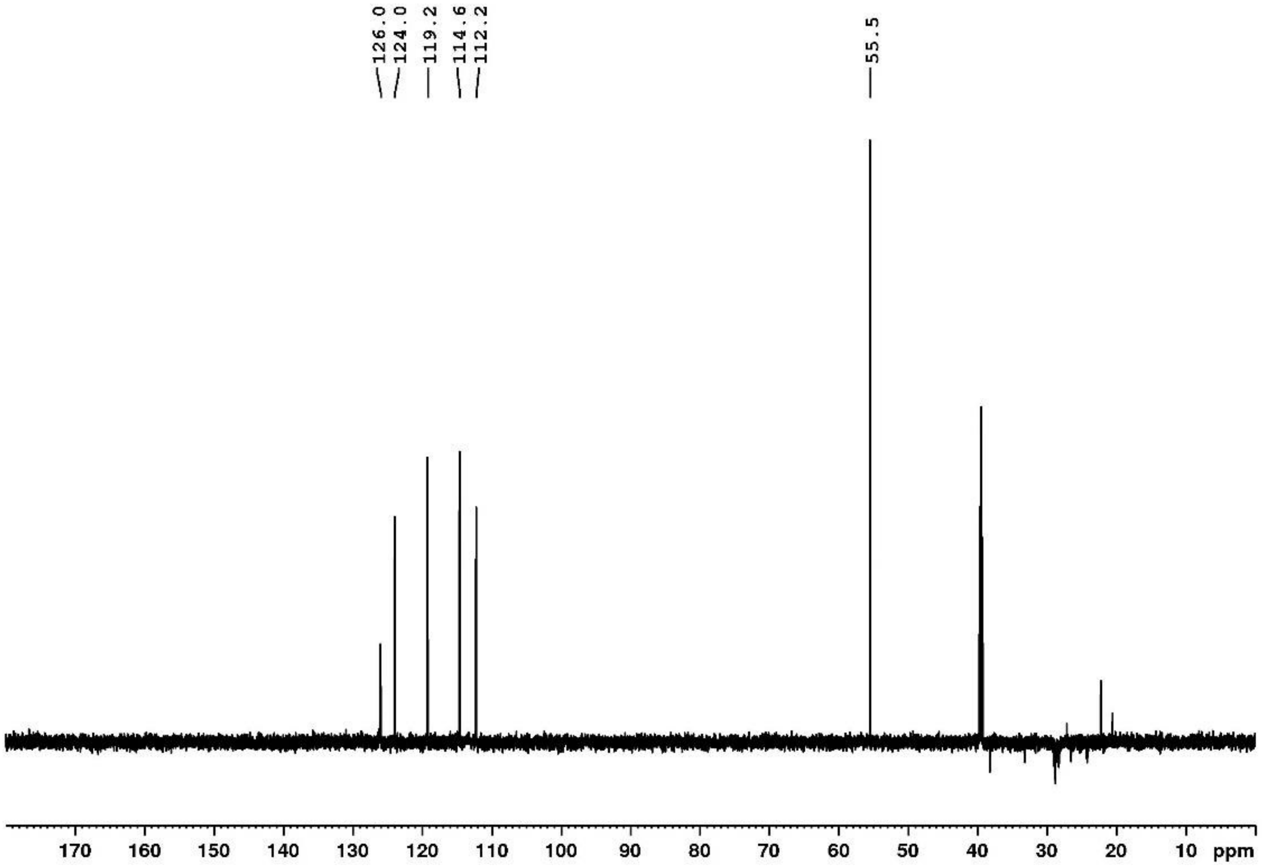

Figure S2. The spectroscopic data of **1**

(E) The HSQC spectrum of compound **1**

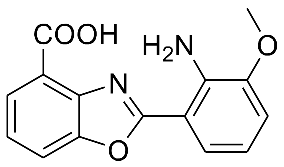

1

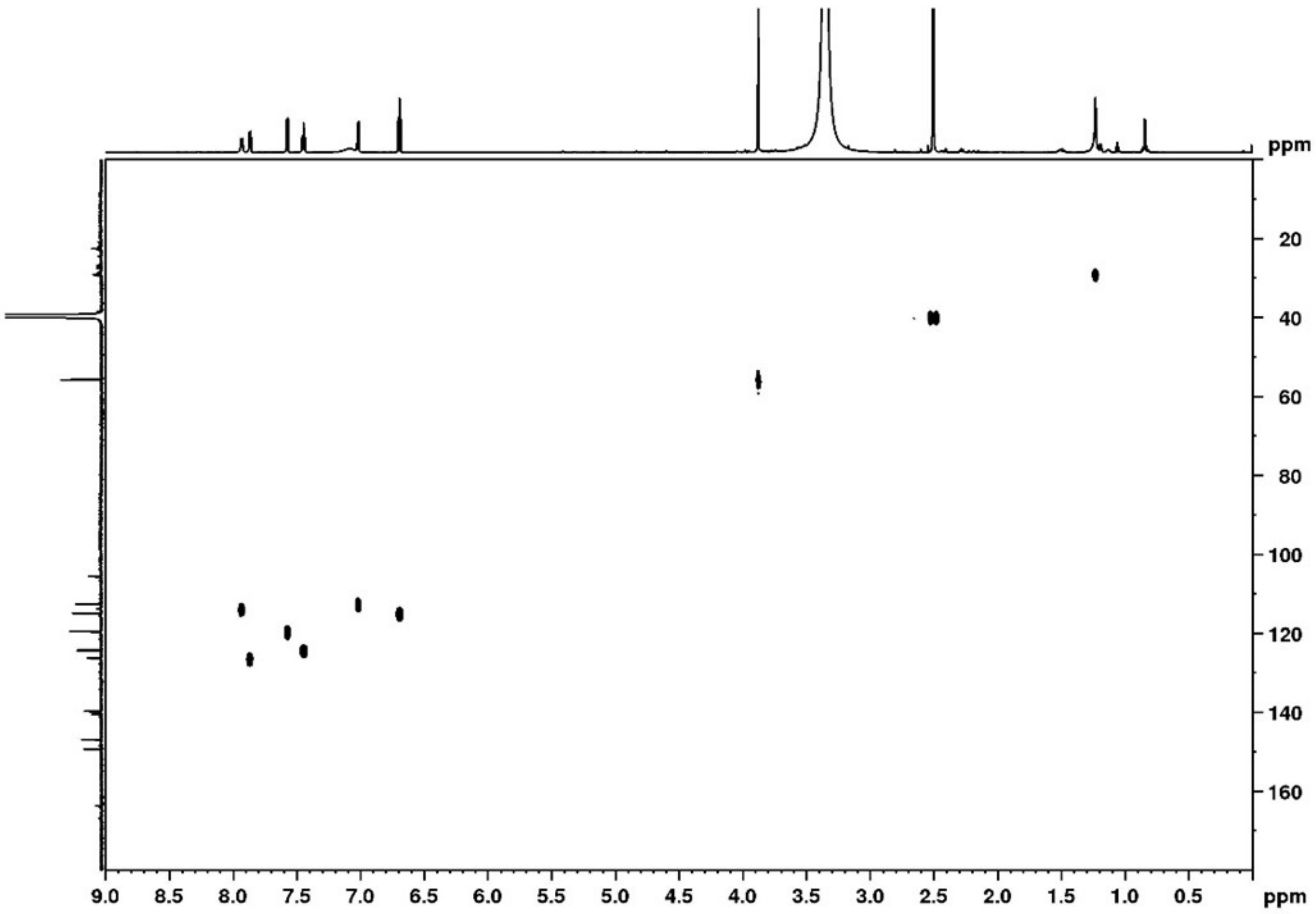

Figure S2. The spectroscopic data of **1**

(F) The HMBC spectrum of **1**

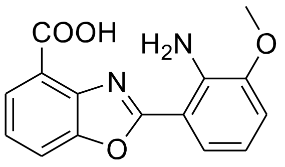

1

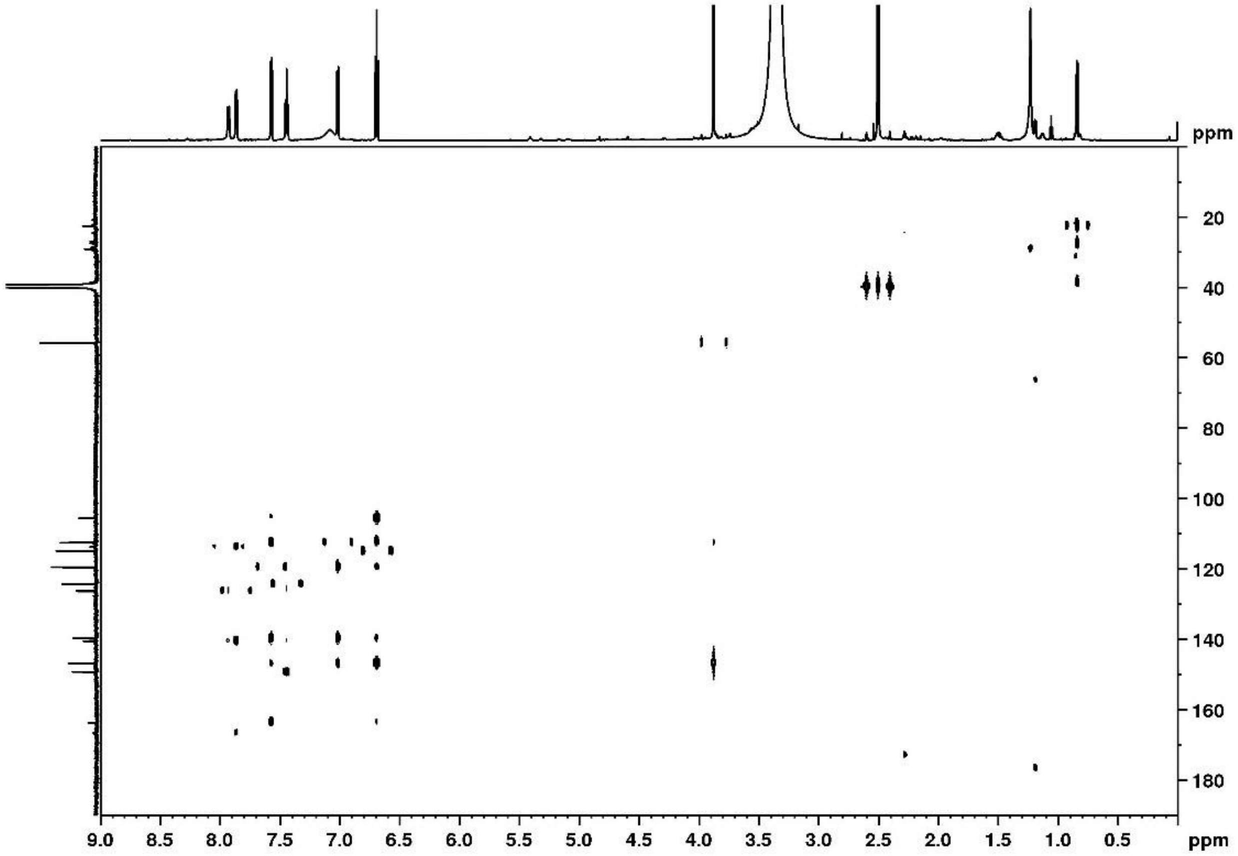

Figure S2. The spectroscopic data of **1**

(G) The ^{1}H-^{1}H COSY spectrum of **1**

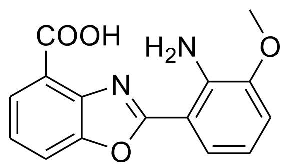

1

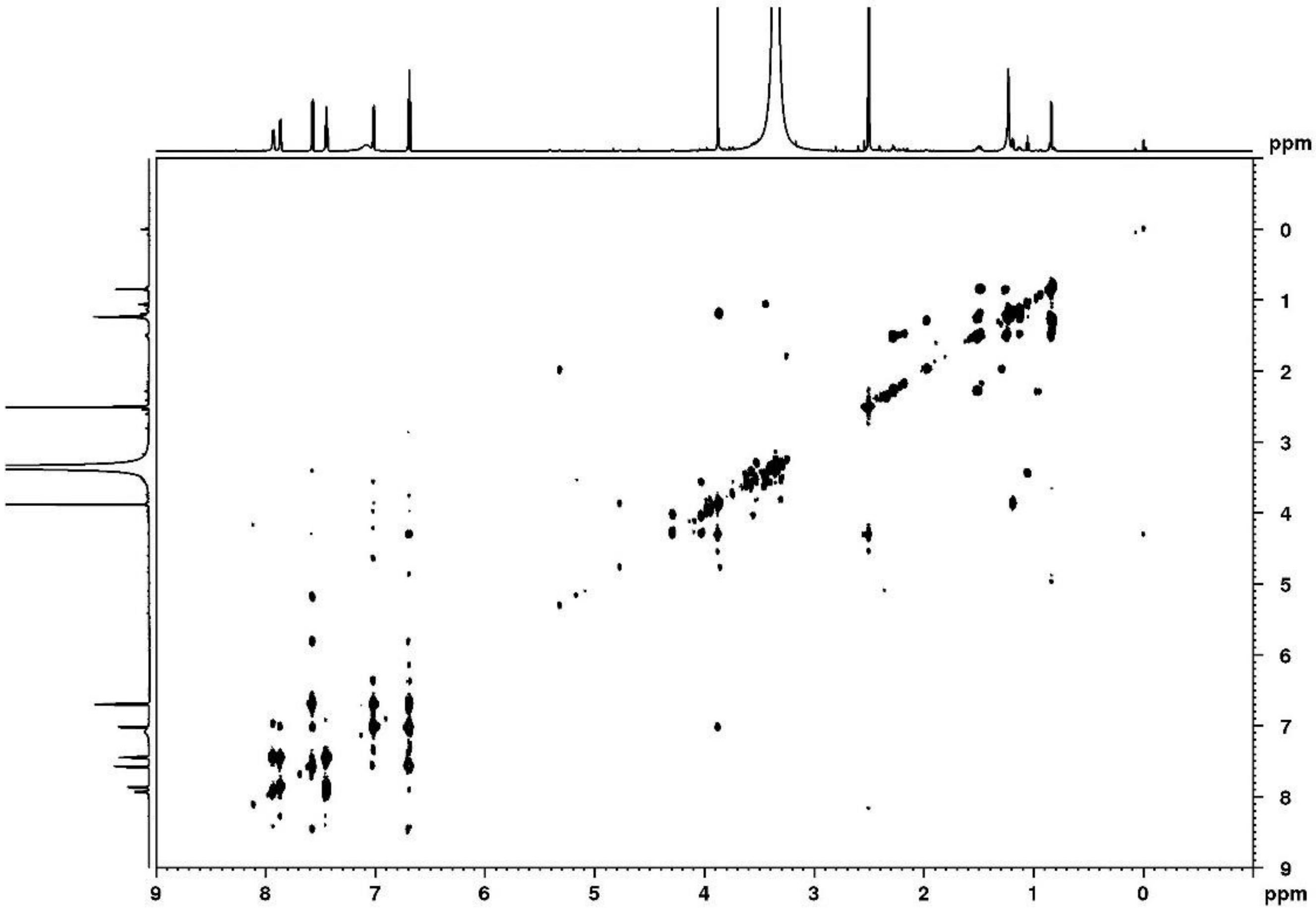

Figure S3. The spectroscopic data of **2**

(A) The HRESIMS (a), UV (b), CD (c) spectrum and chiral HPLC analysis (d) of **2**

(a)

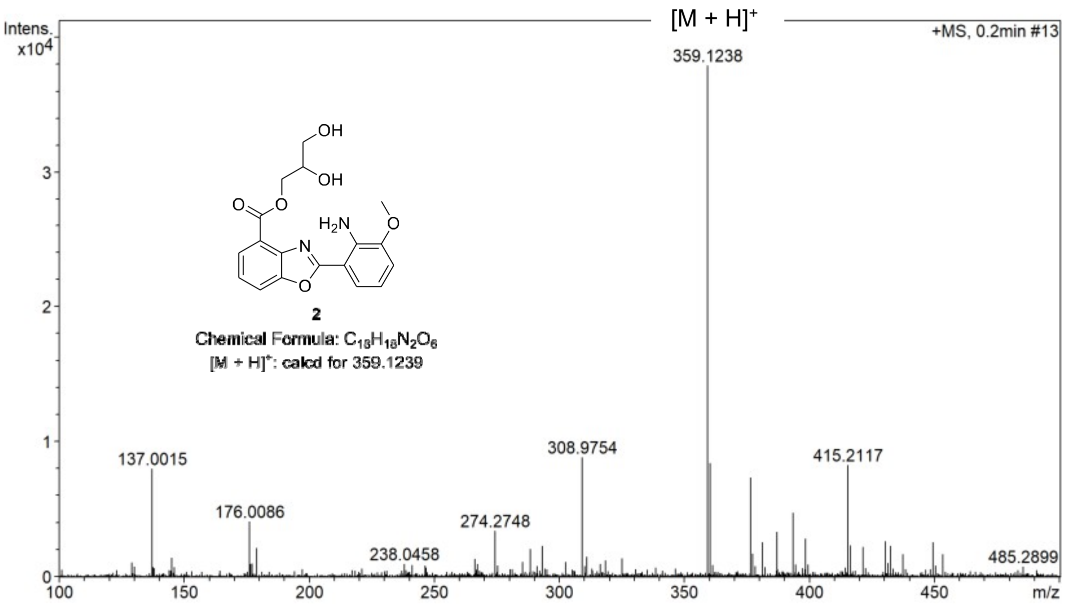

(b)

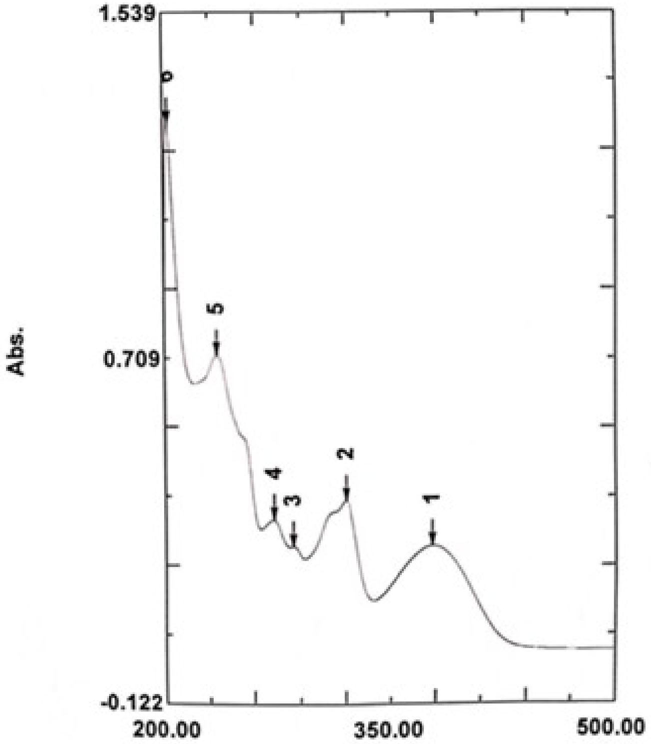

(c)

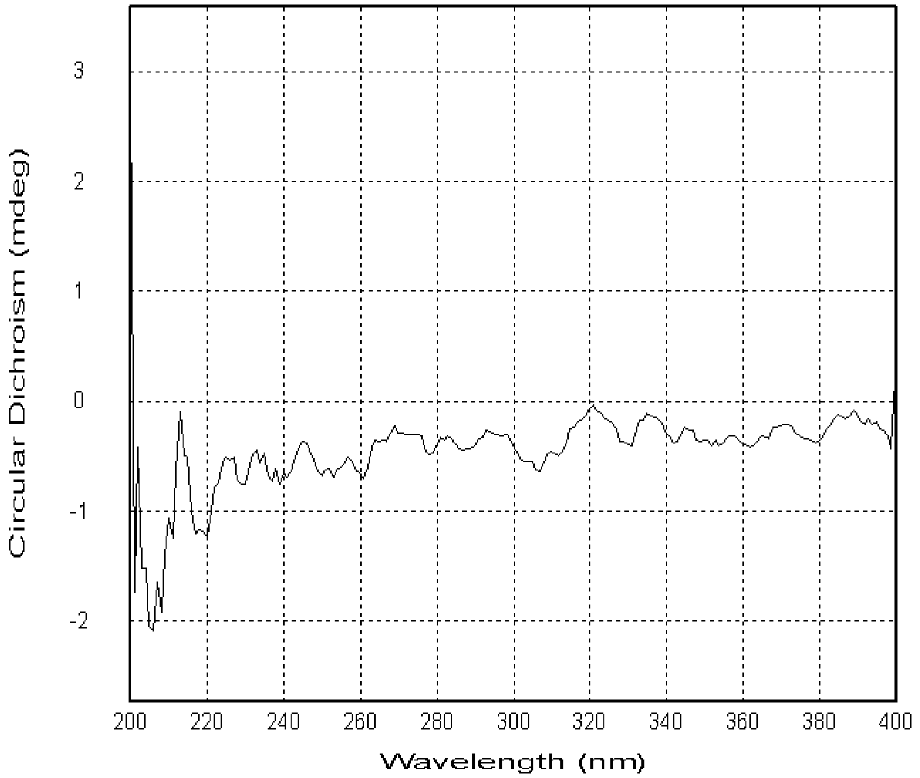

(d) Chiral HPLC analysis of compound **2**

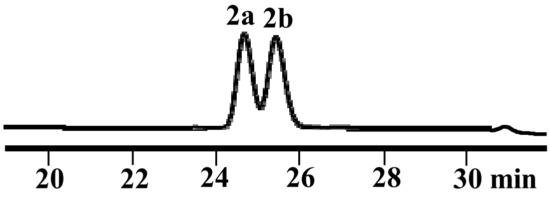

Chiral HPLC analysis of compound **2** was conducted by using a chiral column (Lux Cellulose-3, 5 μm, 250 × 4.6 mm, phenomenex) with UV detection at 265 nm under the following program: solvent system (solvent A, 10% CH_3CN in water; solvent B, 90% CH_3CN in water); 38% B (0–35 min), flow rate at 1 mL min^{-1}.

Figure S3. The spectroscopic data of **2**

(B) The ^{1}H-NMR spectrum of **2** (700 MHz for ^{1}H NMR in DMSO-d_6)

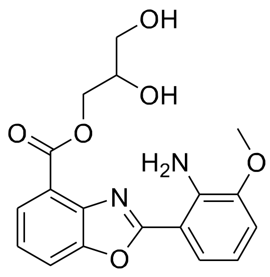

2

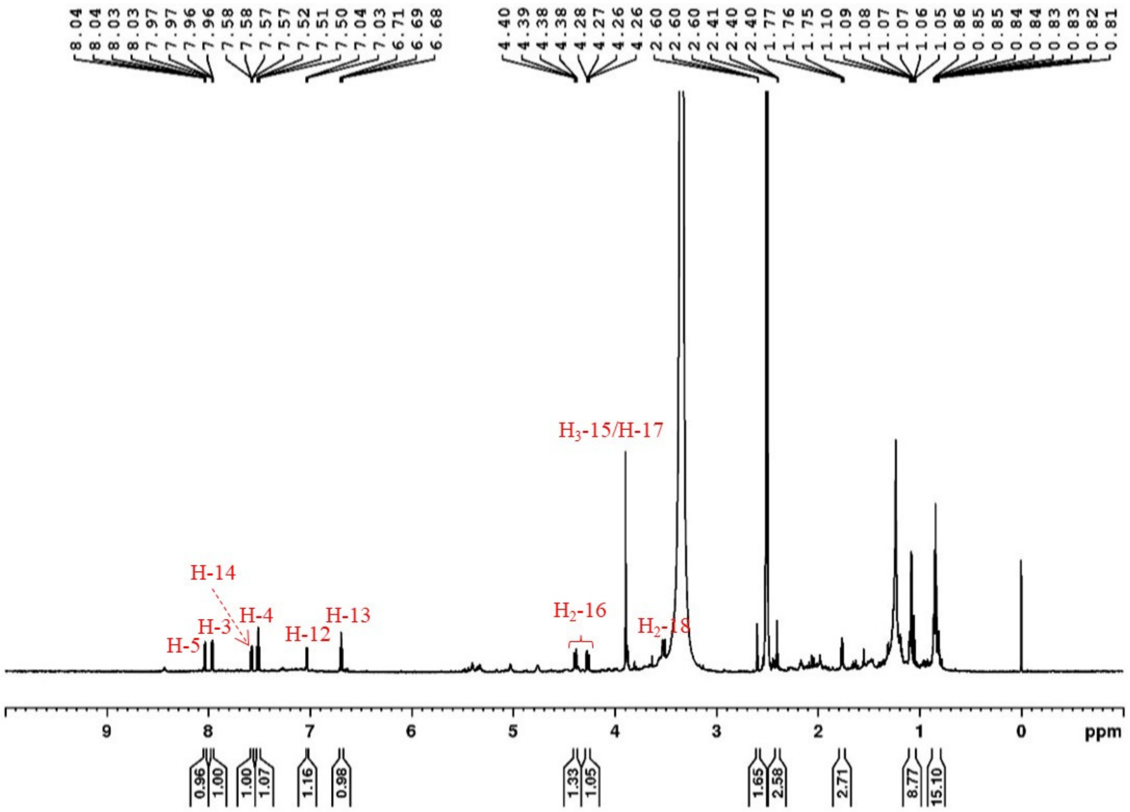

Figure S3. The spectroscopic data of **2**

(C) The ^{13}C NMR spectrum of compound **2** in DMSO-d_6 (175 MHz)

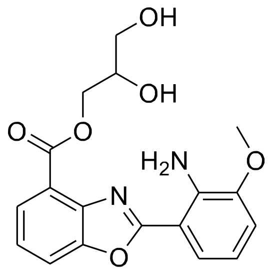

2

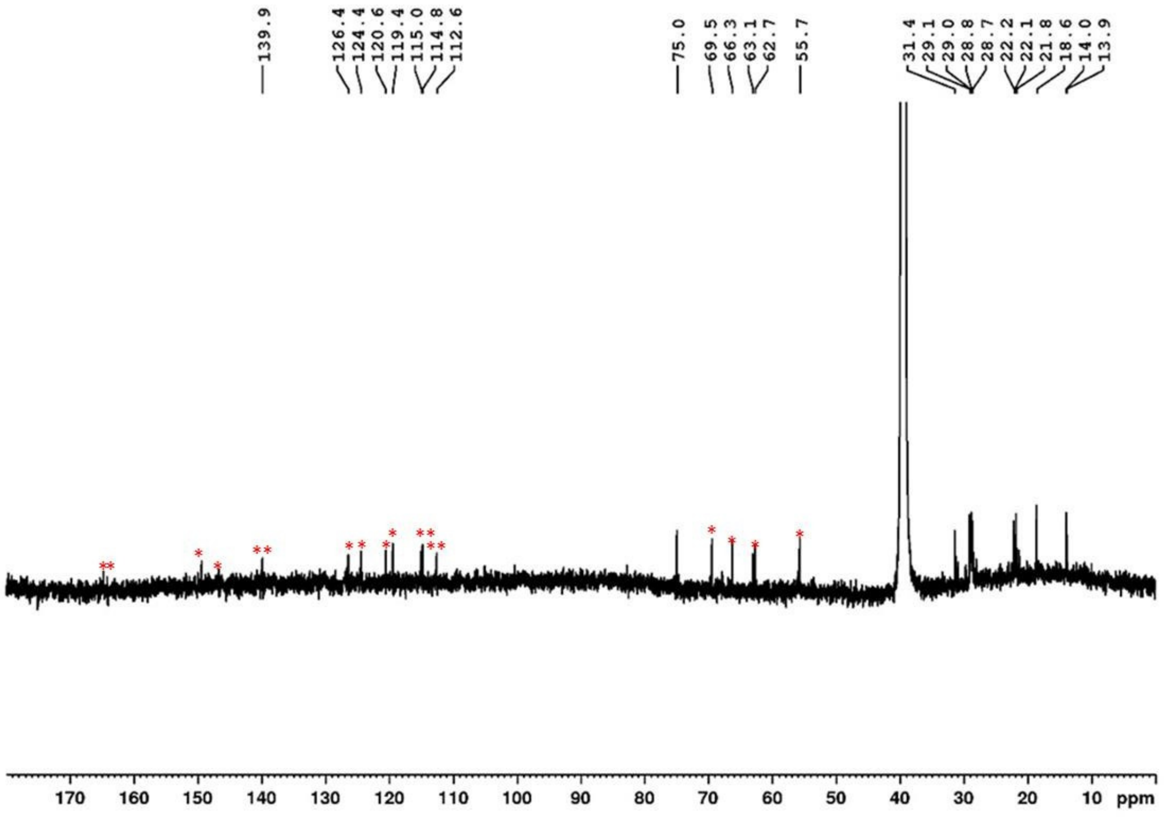

Figure S3. The spectroscopic data of **2**

(D) The DEPT135 spectrum of **2**

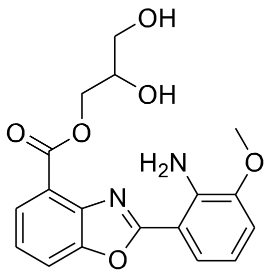

2

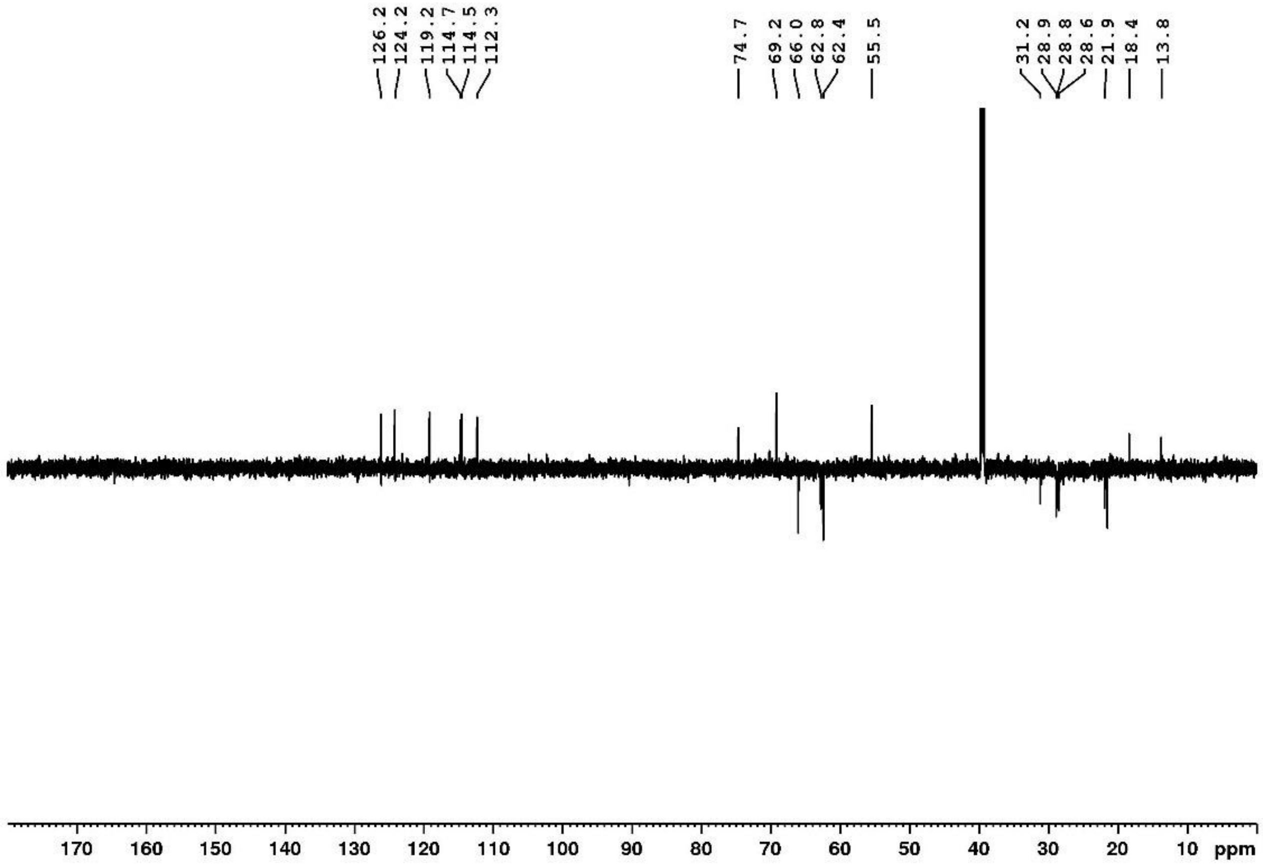

Figure S3. The spectroscopic data of **2**

(E) The HSQC spectrum of compound **2**

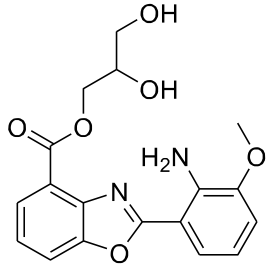

2

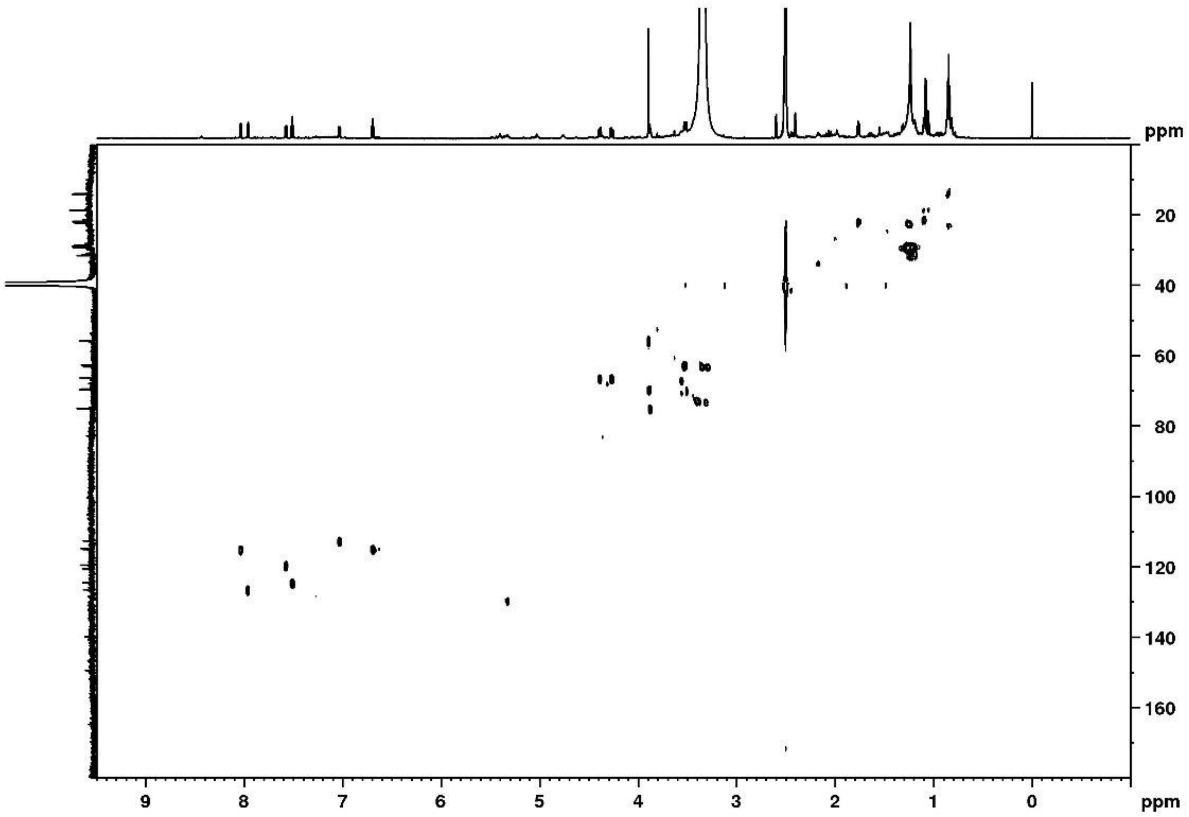

Figure S3. The spectroscopic data of **2**

(F) The HMBC spectrum of **2**

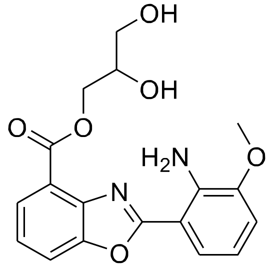

2

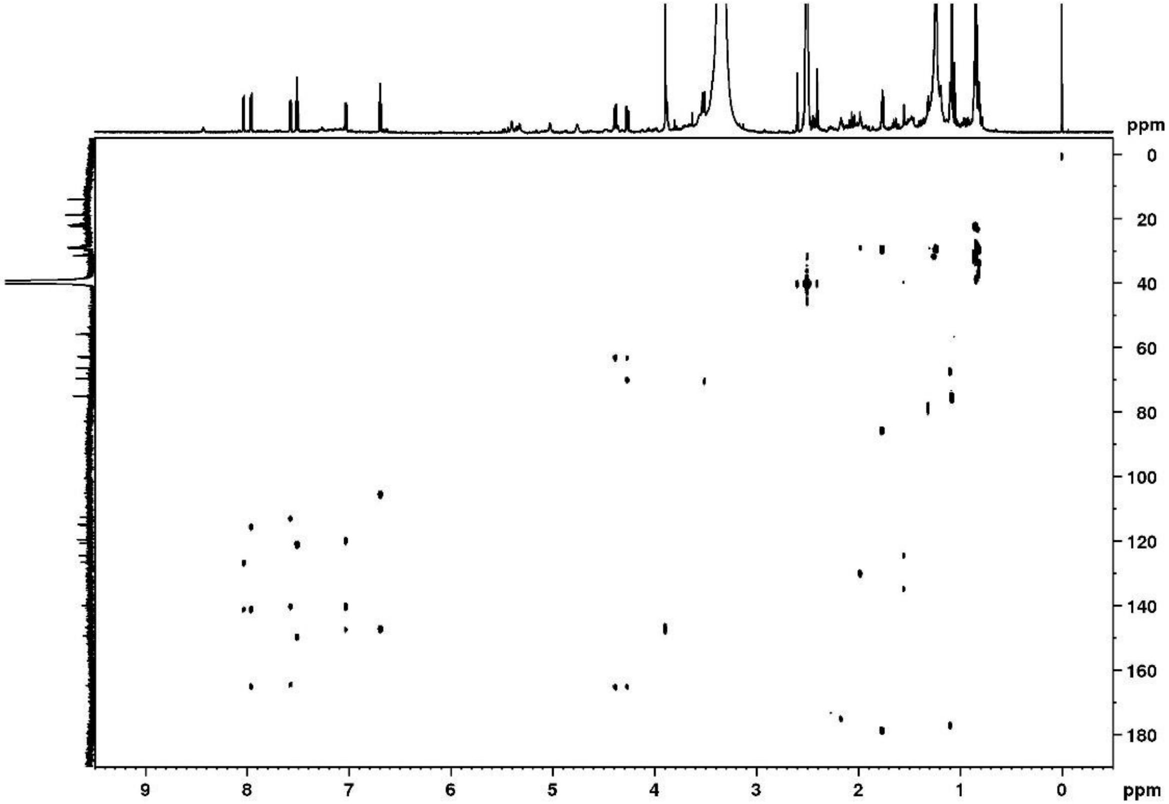

Figure S3. The spectroscopic data of **2**

(G) The ^{1}H-^{1}H COSY spectrum of **2**

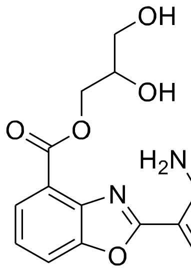

2

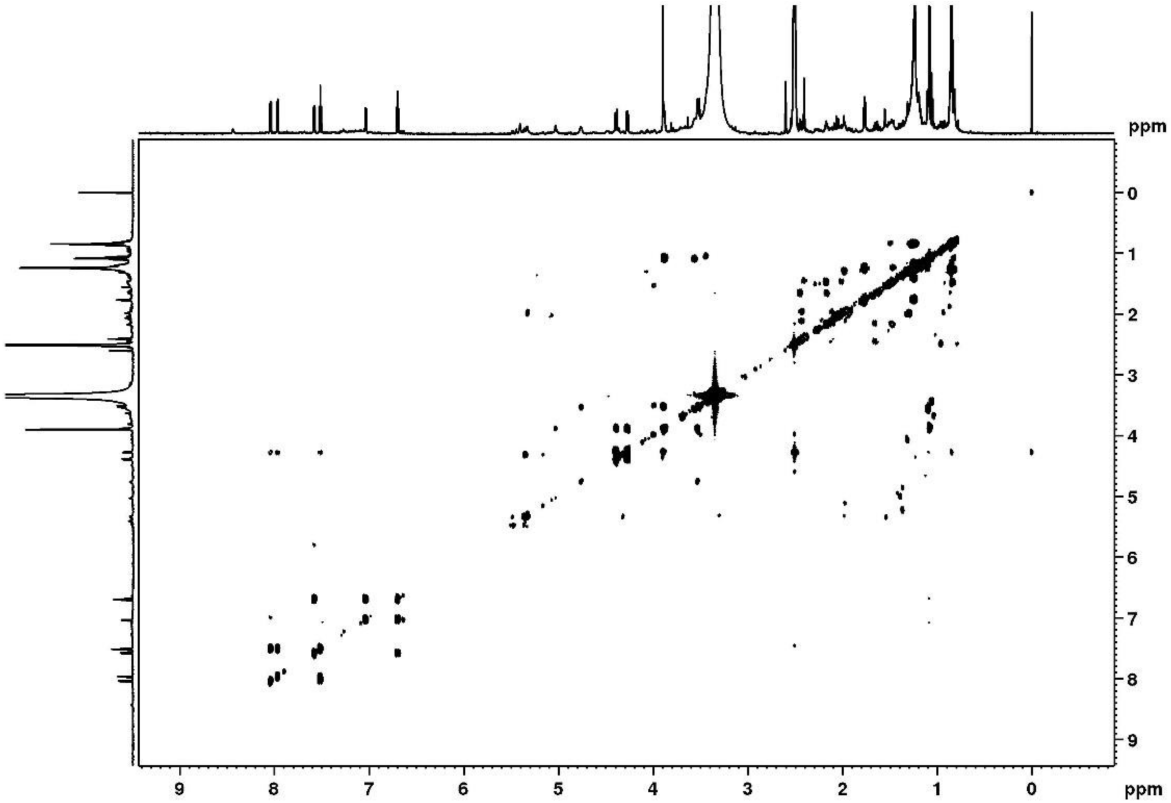

Figure S4. The spectroscopic data of **3**

(A) The HRESIMS (a) UV (b) of **3**

(a)

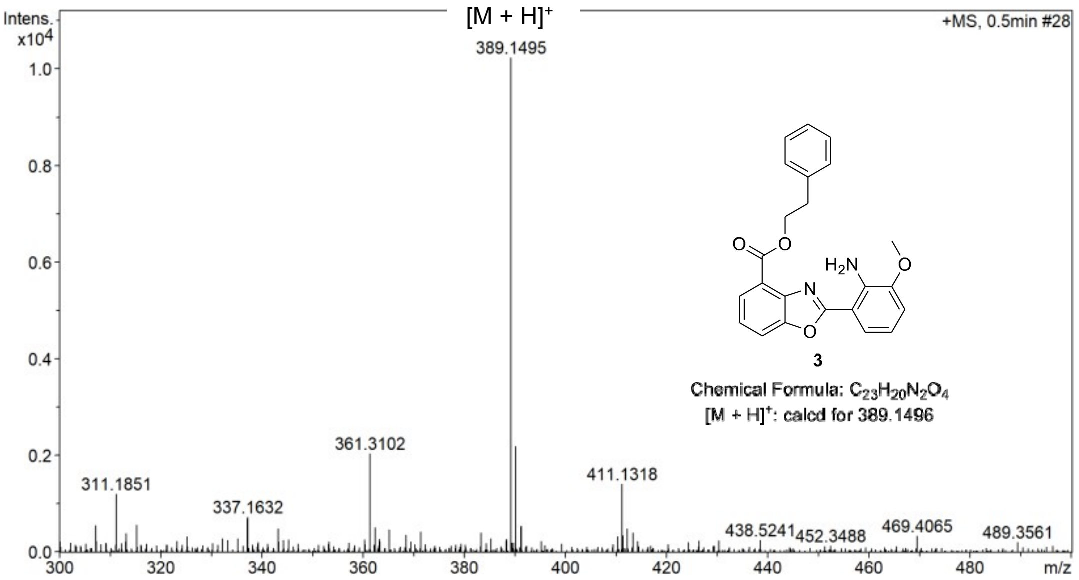

(b)

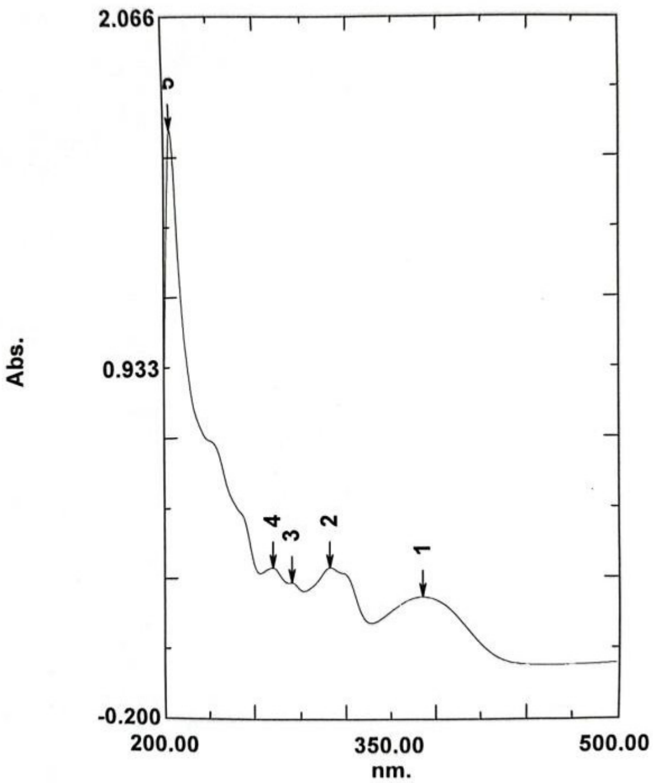

Figure S4. The spectroscopic data of **3**

(B) The ^{1}H-NMR spectrum of **3** (700 MHz for ^{1}H NMR in DMSO-d_6)

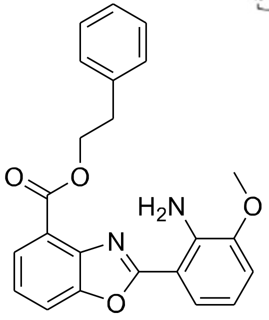

3

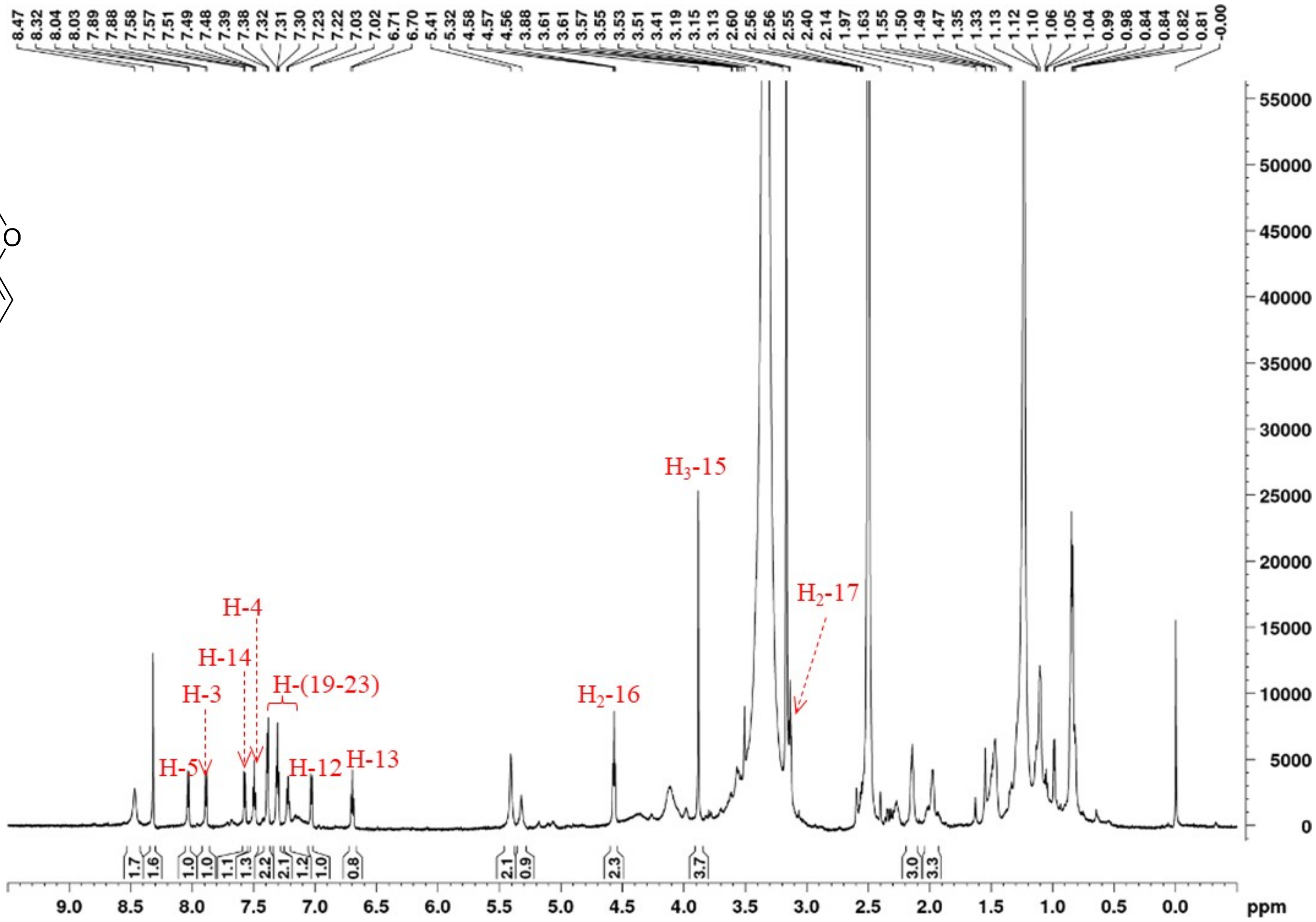

Figure S4. The spectroscopic data of **3**

(C) The^{13}C NMR spectrum of compound **3** in DMSO-d_6 (175 MHz)

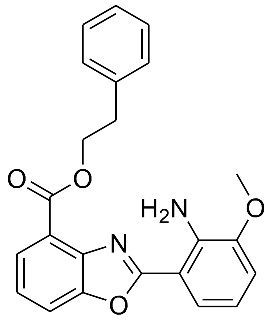

3

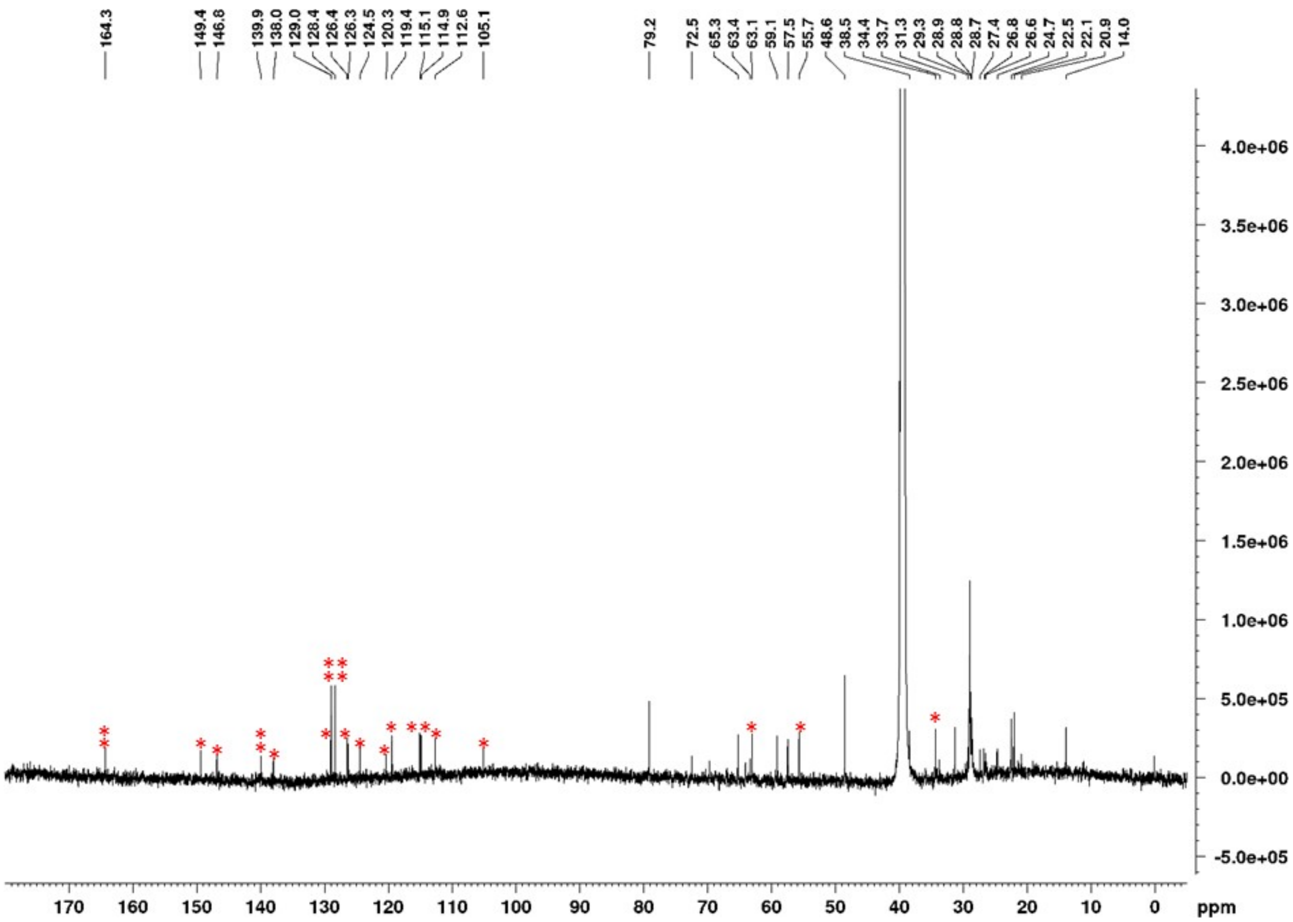

Figure S4. The spectroscopic data of **3**

(D) The DEPT135 spectrum of **3**

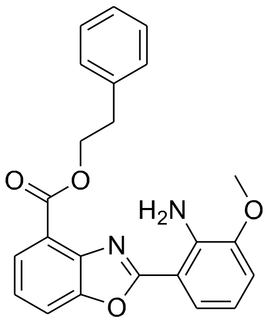

3

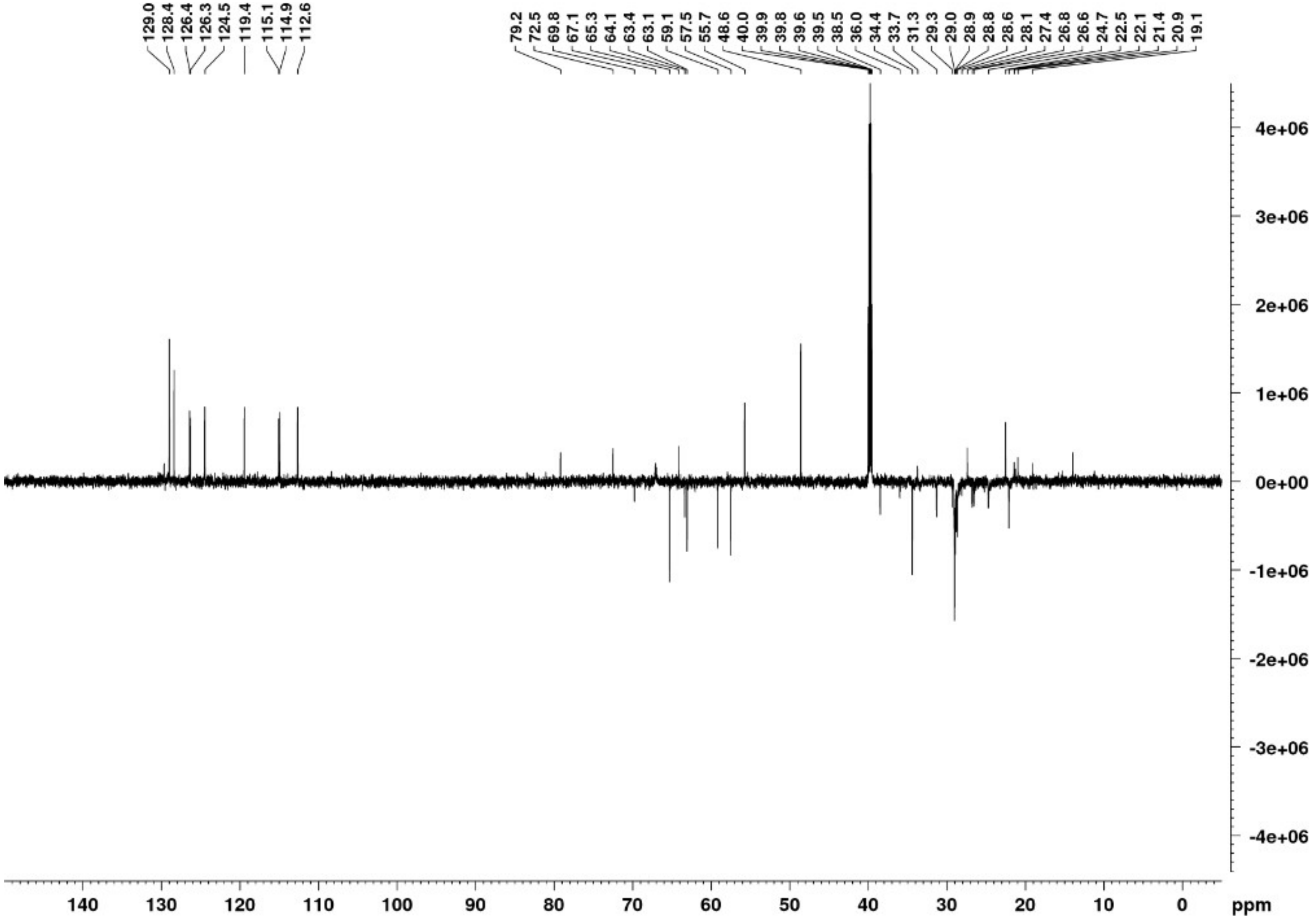

Figure S4. The spectroscopic data of **3**

(E) The HSQC spectrum of compound **3**

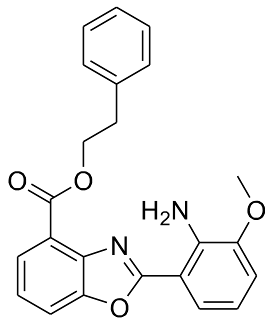

3

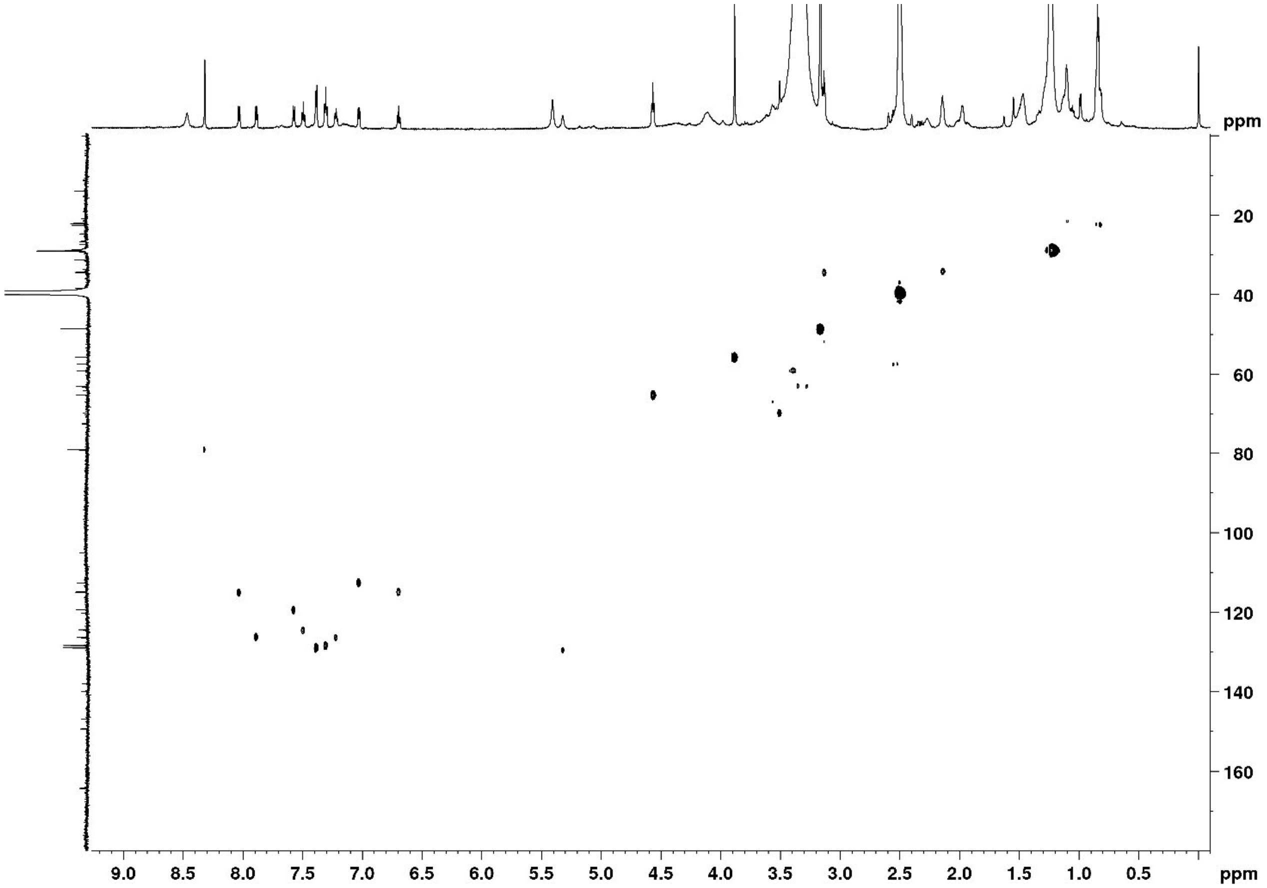

Figure S4. The spectroscopic data of **3**

(F) The HMBC spectrum of **3**

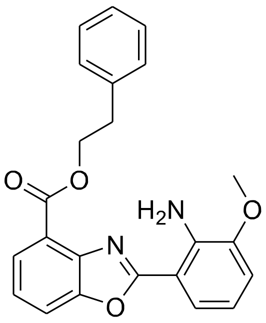

3

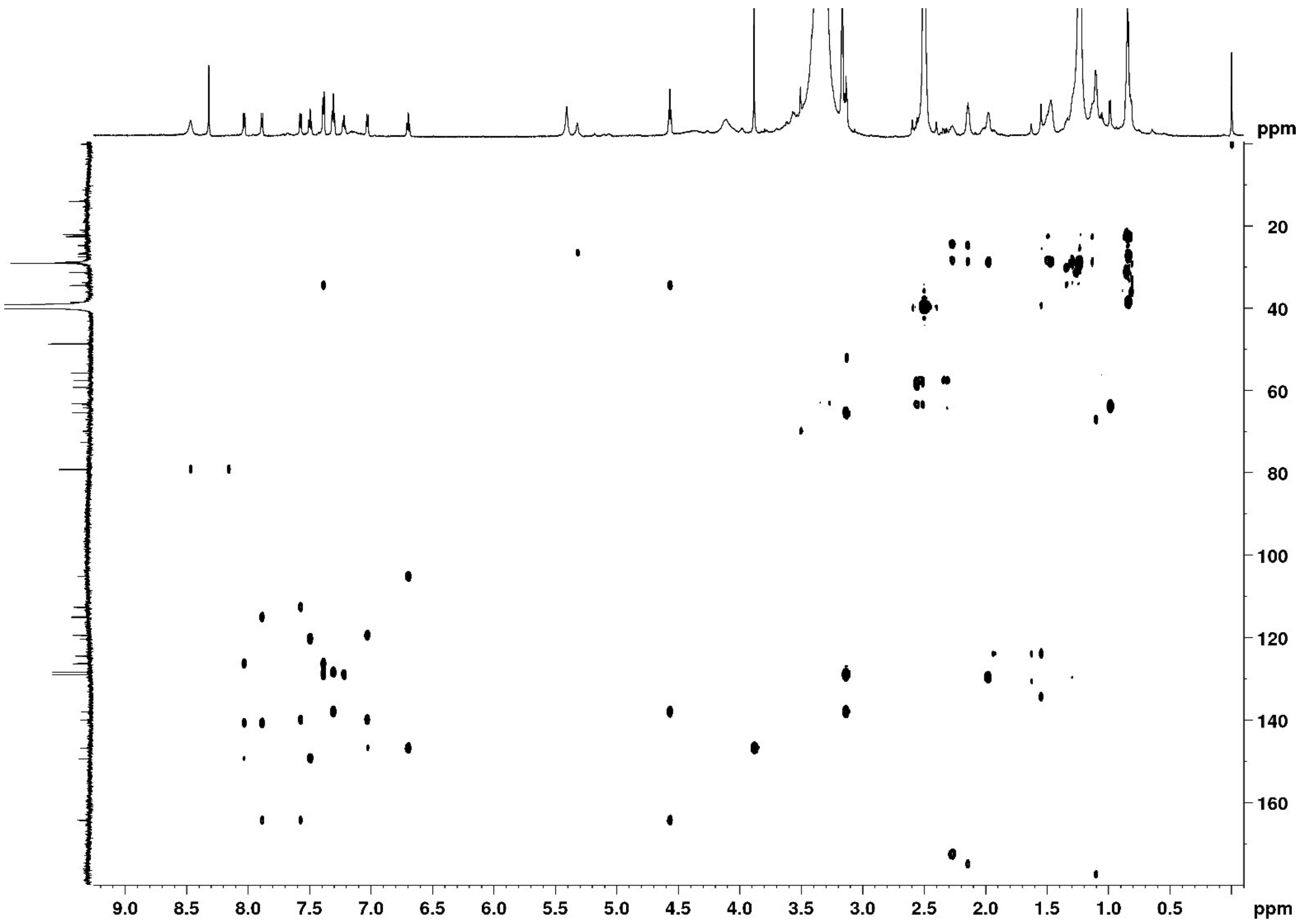

Figure S4. The spectroscopic data of **3**

(G) The ^{1}H-^{1}H COSY spectrum of **3**

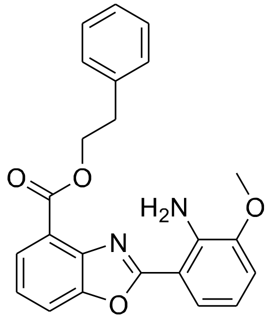

3

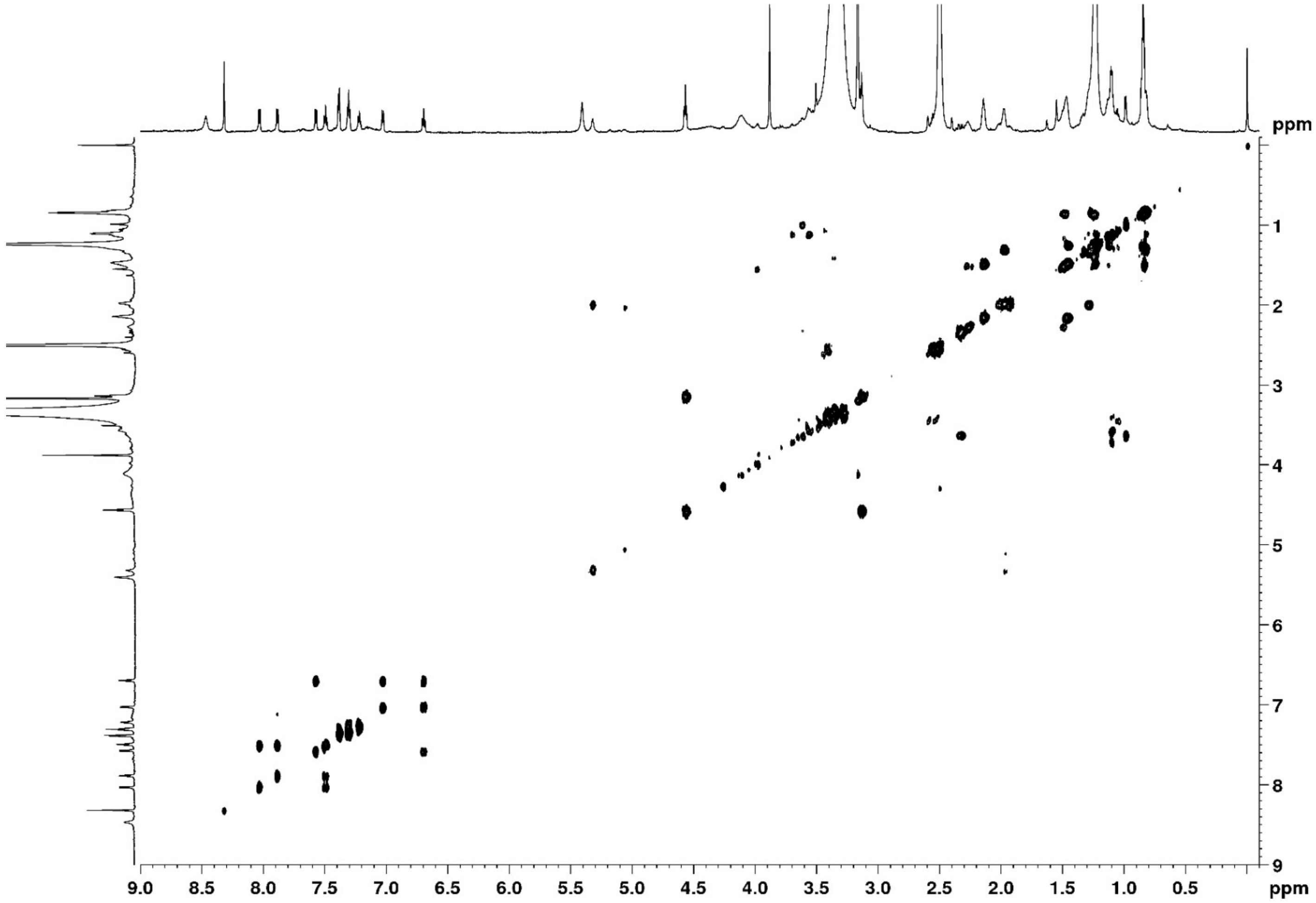

Figure S5. The spectroscopic data of **4**

(A) The HRESIMS (a) and UV (b) spectrum of **4**

(a)

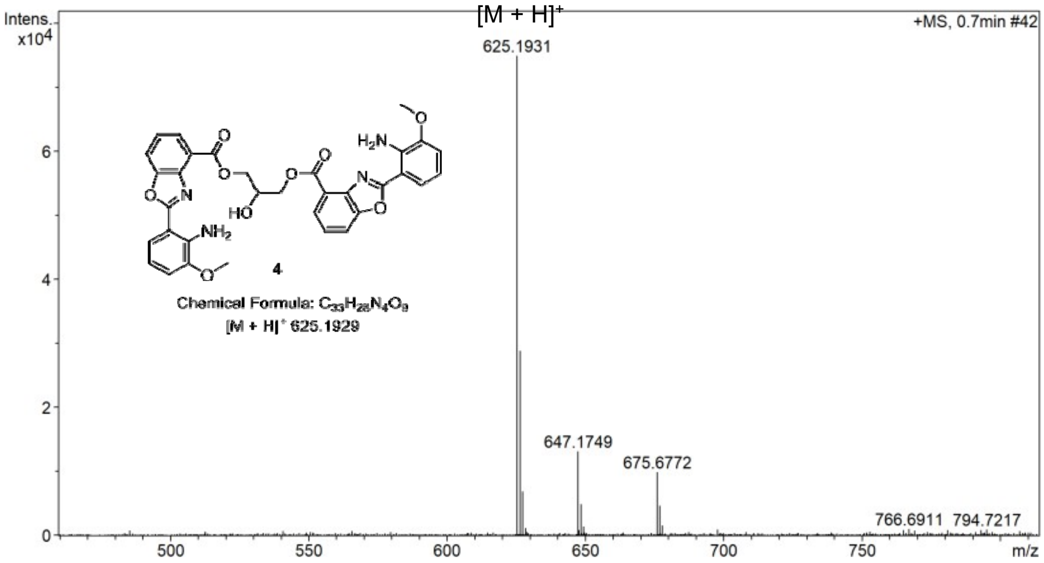

(b)

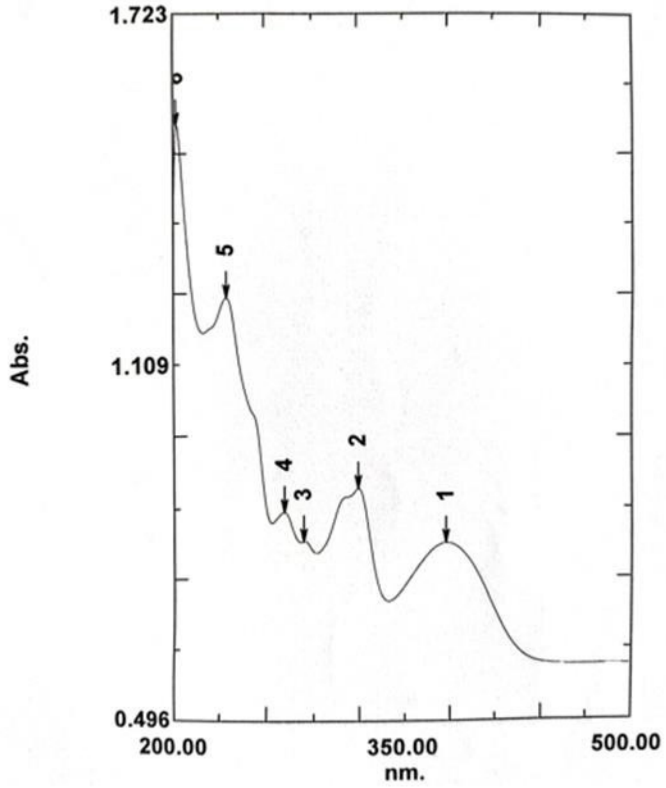

Figure S5. The spectroscopic data of **4**

(B) The ^{1}H-NMR spectrum of **4** (700 MHz for ^{1}H NMR in $CDCl_3$)

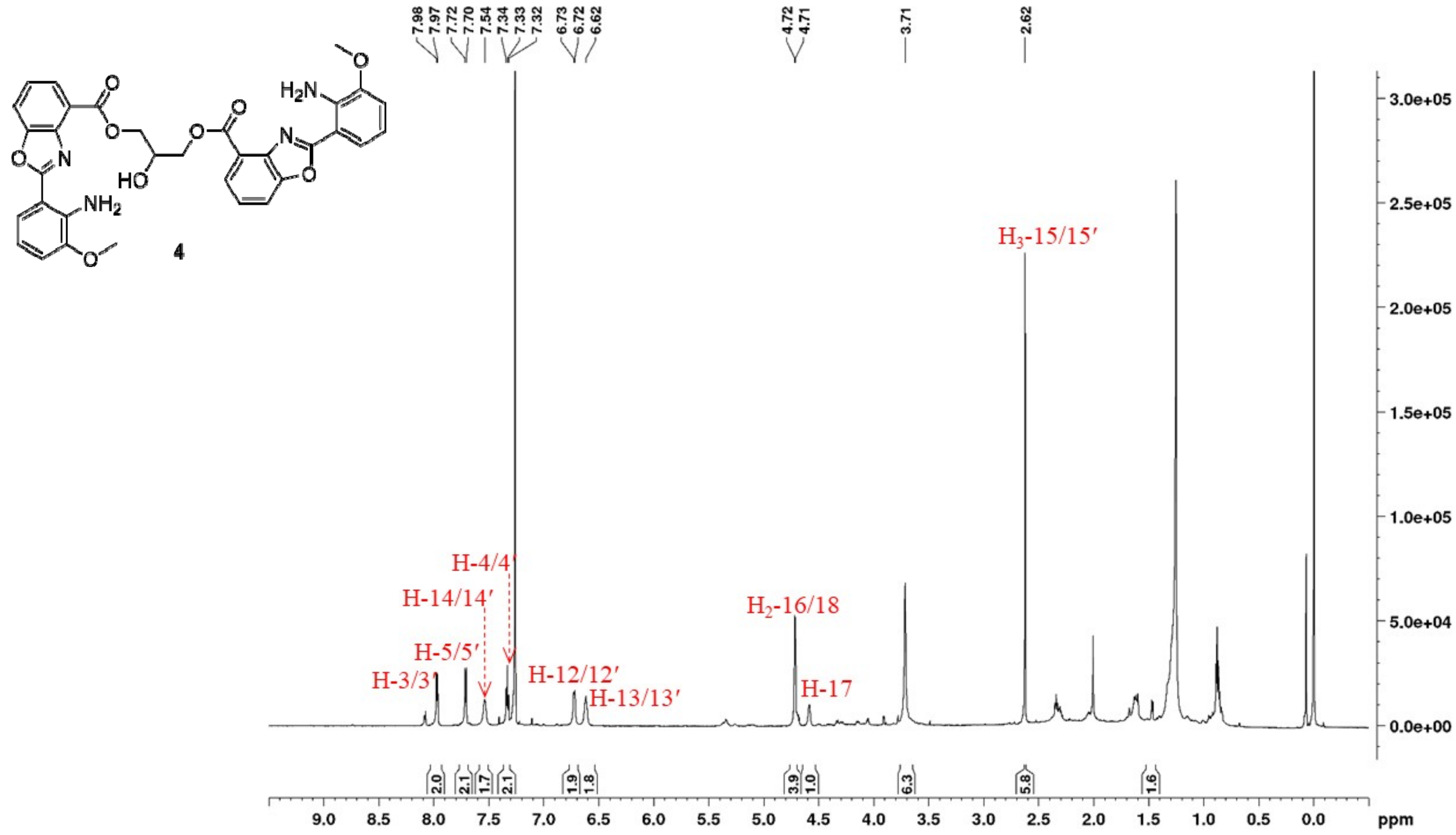

Figure S5. The spectroscopic data of **4**

(C) The ^{13}C NMR spectrum of compound **4** in $CDCl_3$ (175 MHz)

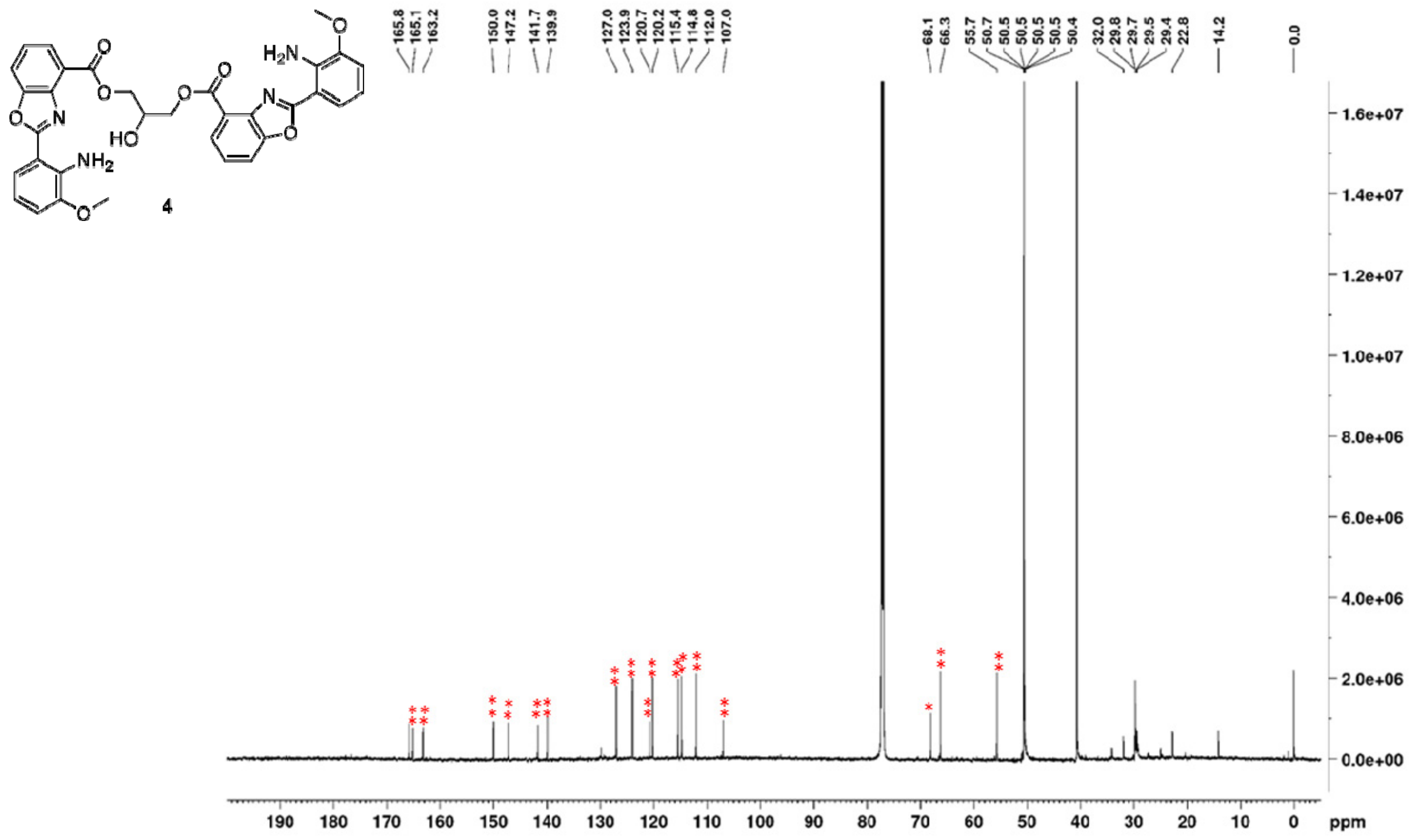

Figure S5. The spectroscopic data of **4**

(D) The DEPT135 spectrum of **4**

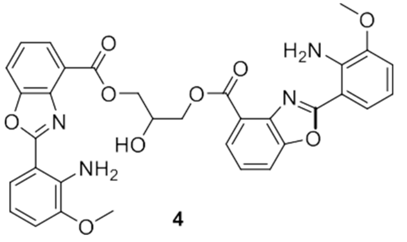

4

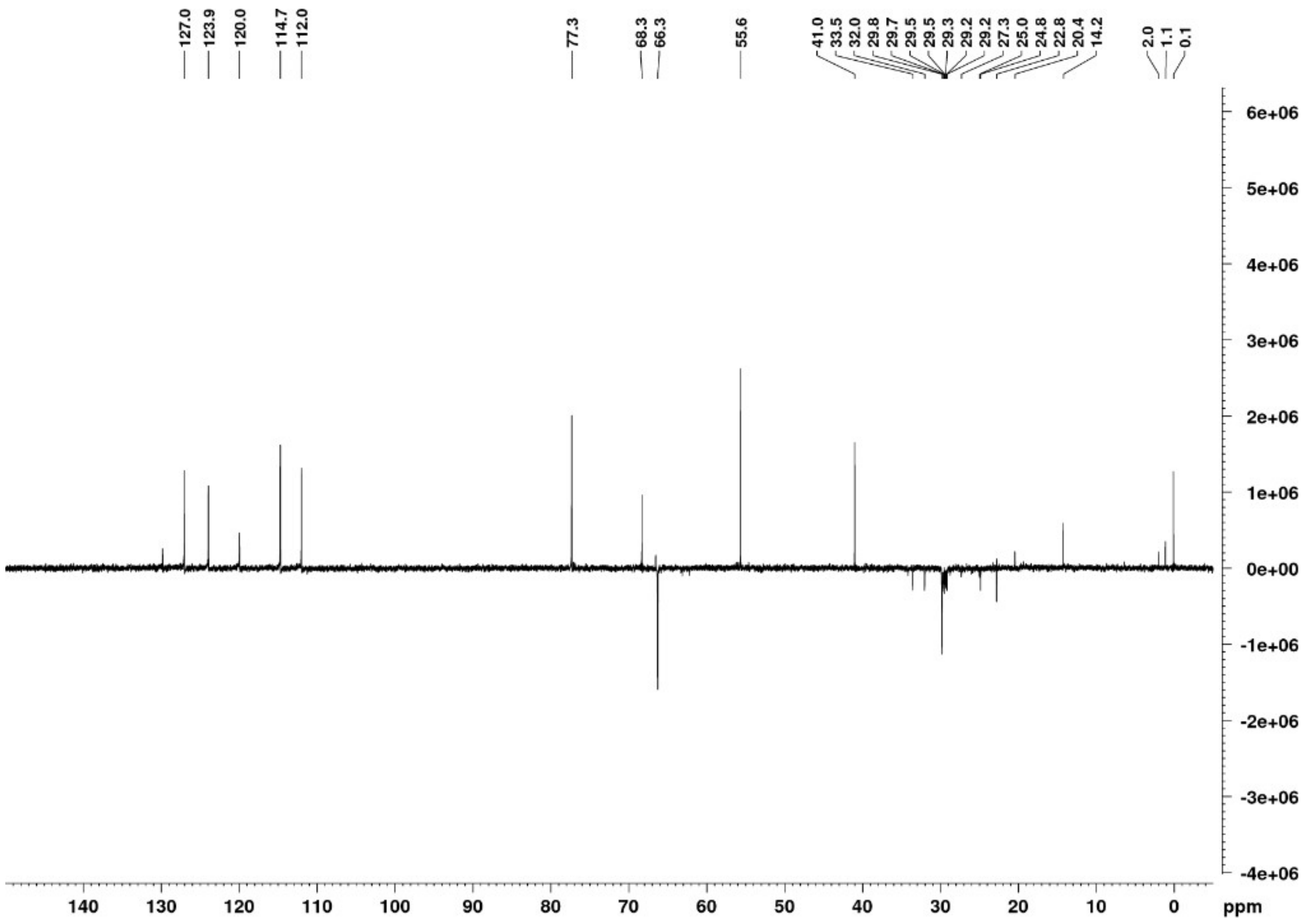

Figure S5. The spectroscopic data of **4**

(E) The HSQC spectrum of compound **4**

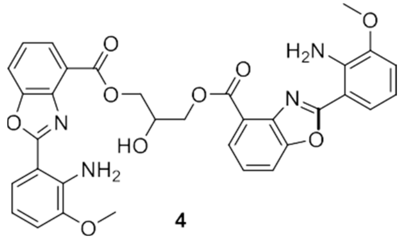

4

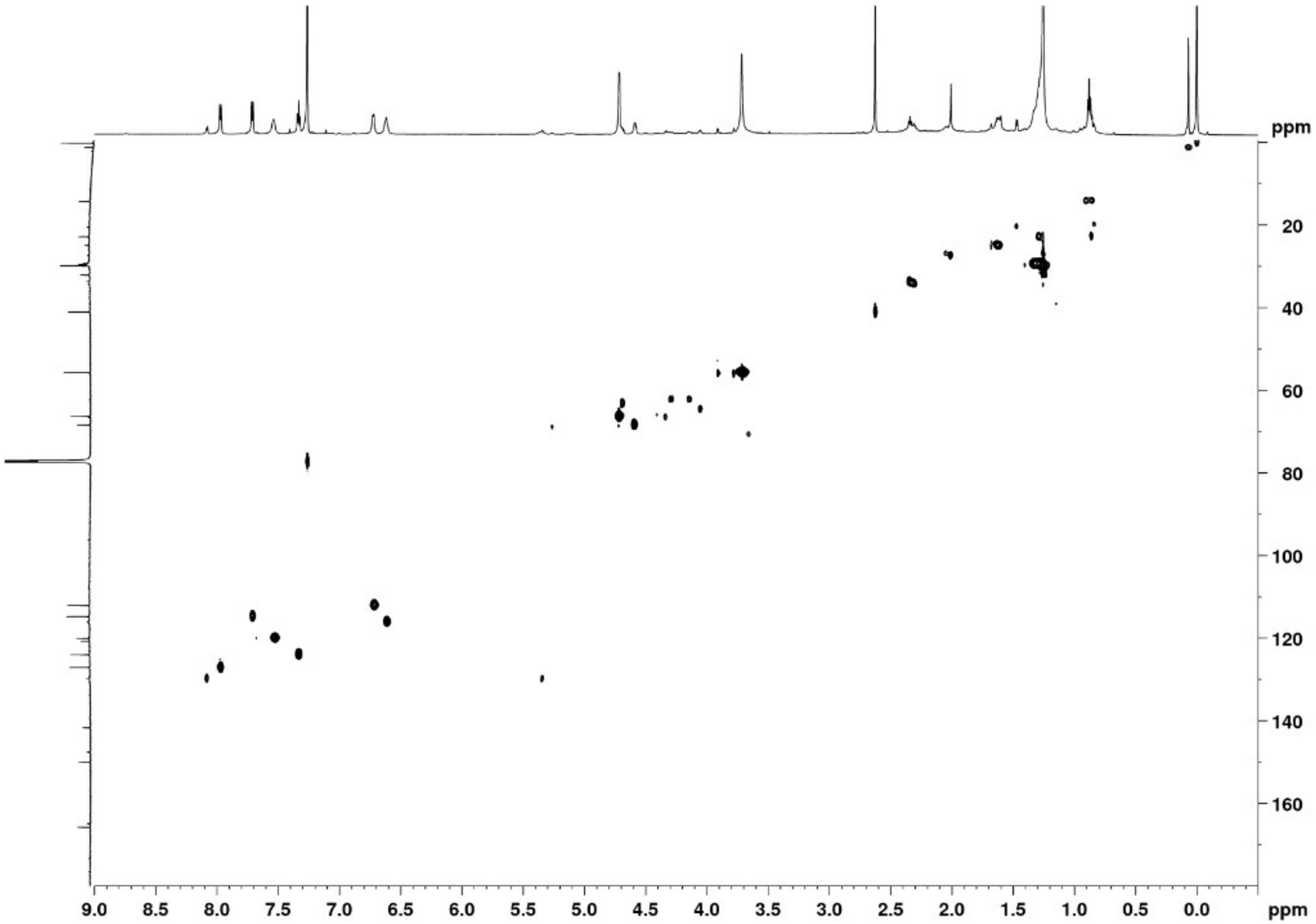

Figure S5. The spectroscopic data of **4**

(F) The HMBC spectrum of **4**

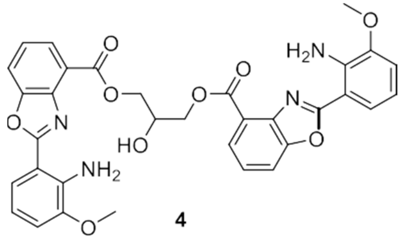

4

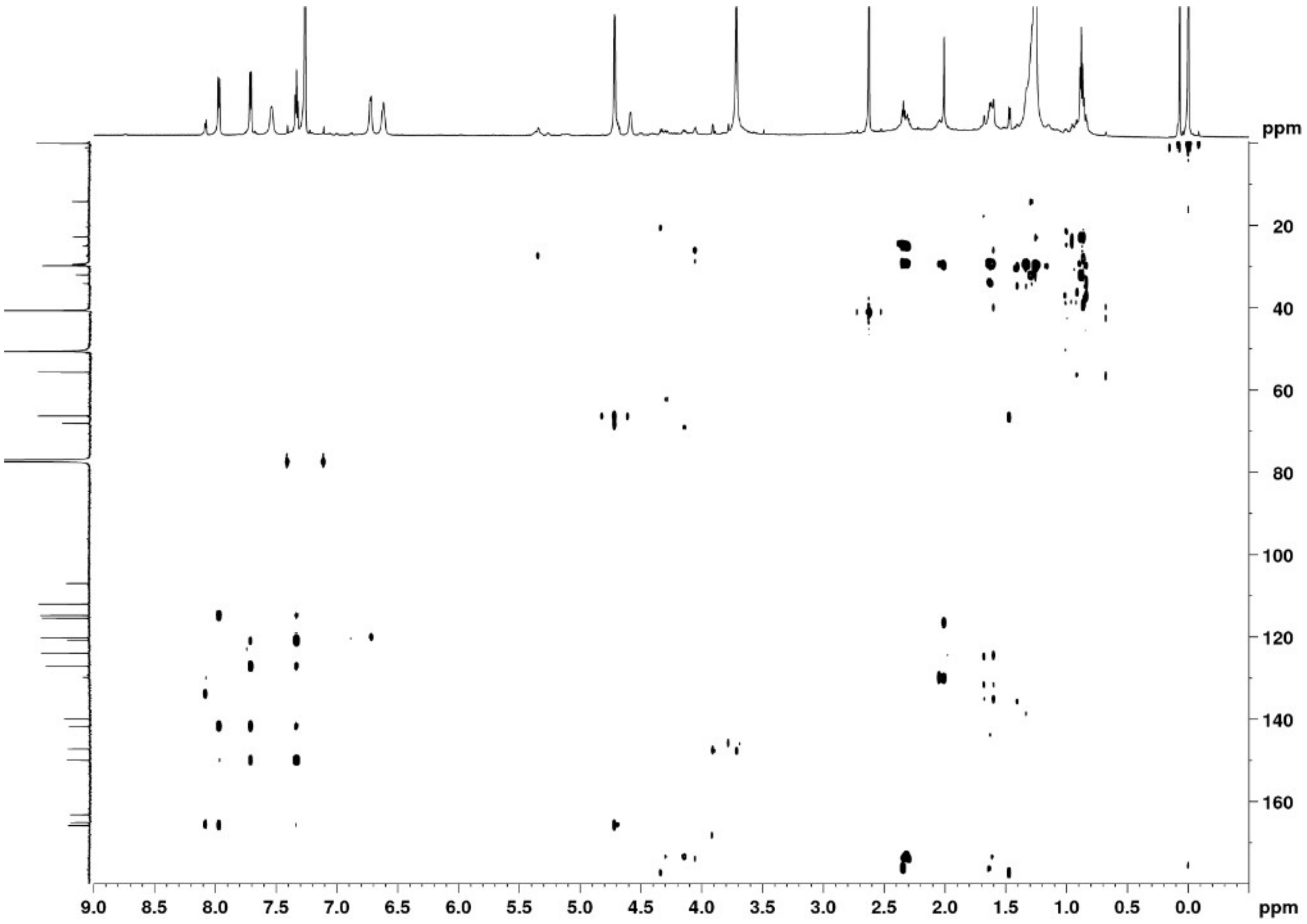

Figure S5. The spectroscopic data of **4**

(G) The ^{1}H-^{1}H COSY spectrum of **4**

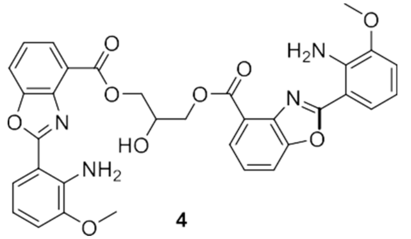

4

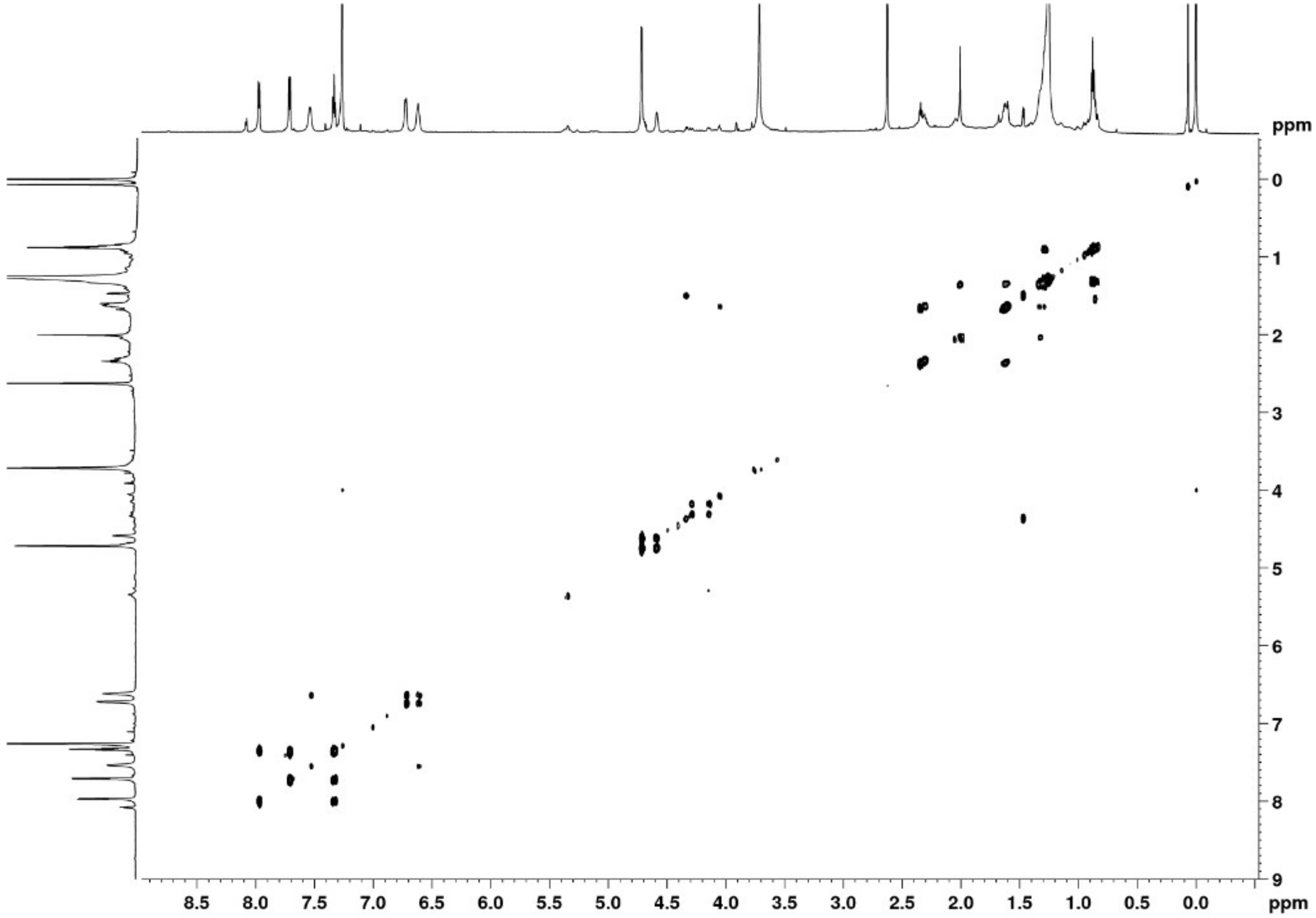

Figure S6. The spectroscopic data of **5**

(A) The HRESIMS (a), UV (b), CD (c) spectrum and chiral HPLC analysis (d) of **5**

(a)

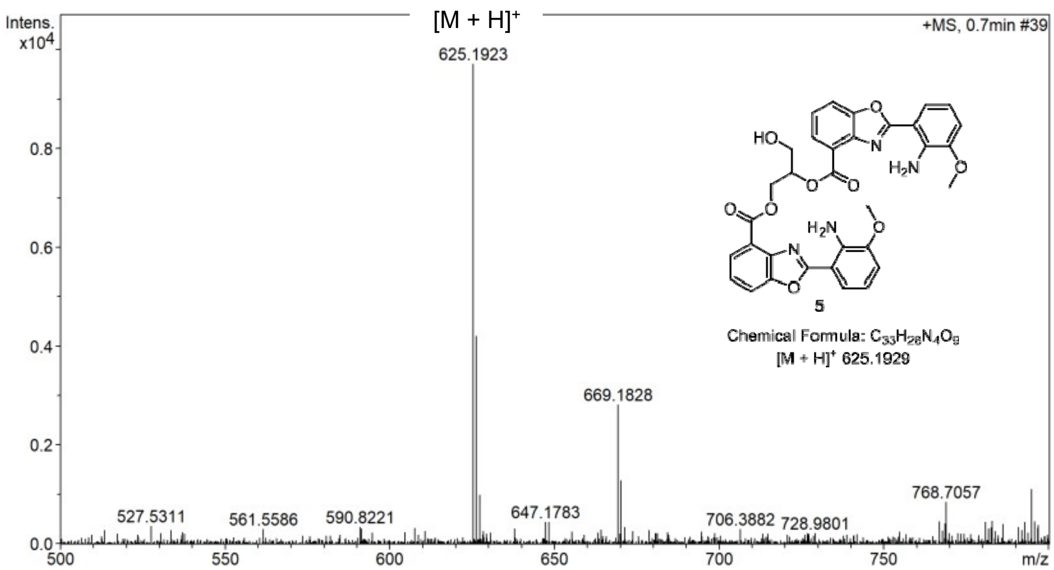

(b)

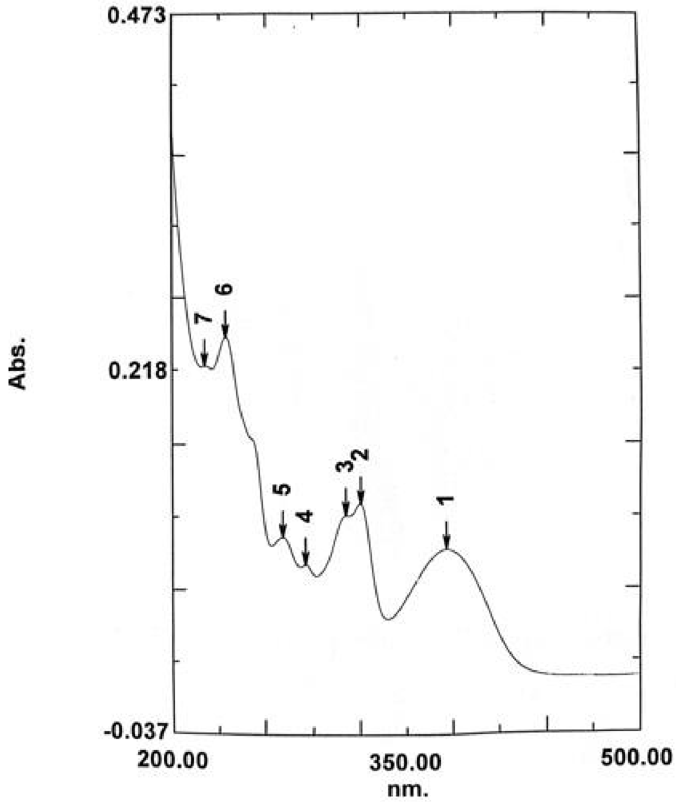

(c)

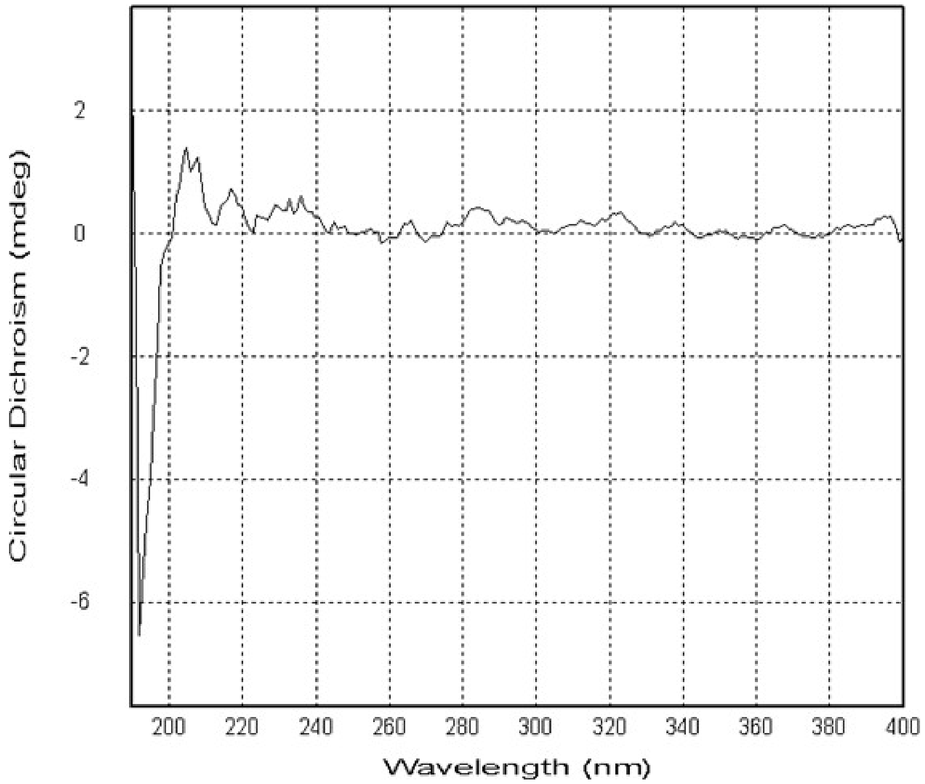

(d) Chiral HPLC analysis of **5**

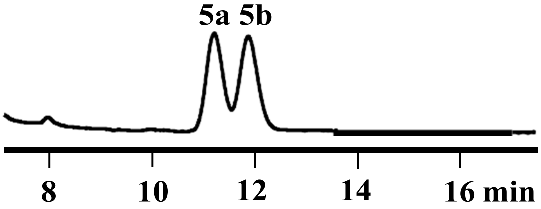

Chiral HPLC analysis of compound **5** was conducted by using a chiral column (Lux Cellulose-4, 5 μm, 250 × 4.6 mm, phenomenex) using the following program: solvent system (solvent A, 10% CH_3CN in water; solvent B, 90% CH_3CN in water); 5% B to 100% B (0–20 min), 100% B (21–25 min), 100% B to 5%B (25–26 min), 5% B (26–30 min), flow rate at 1 mL min^{-1}.

Figure S6. The spectroscopic data of **5**

(B) The ^{1}H-NMR spectrum of **5** (700 MHz for 1H NMR in $CDCl_3$)

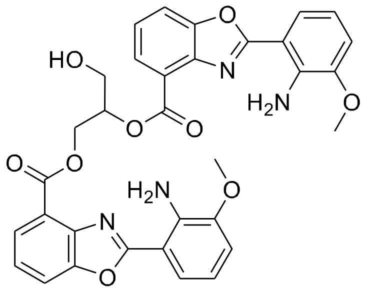

5

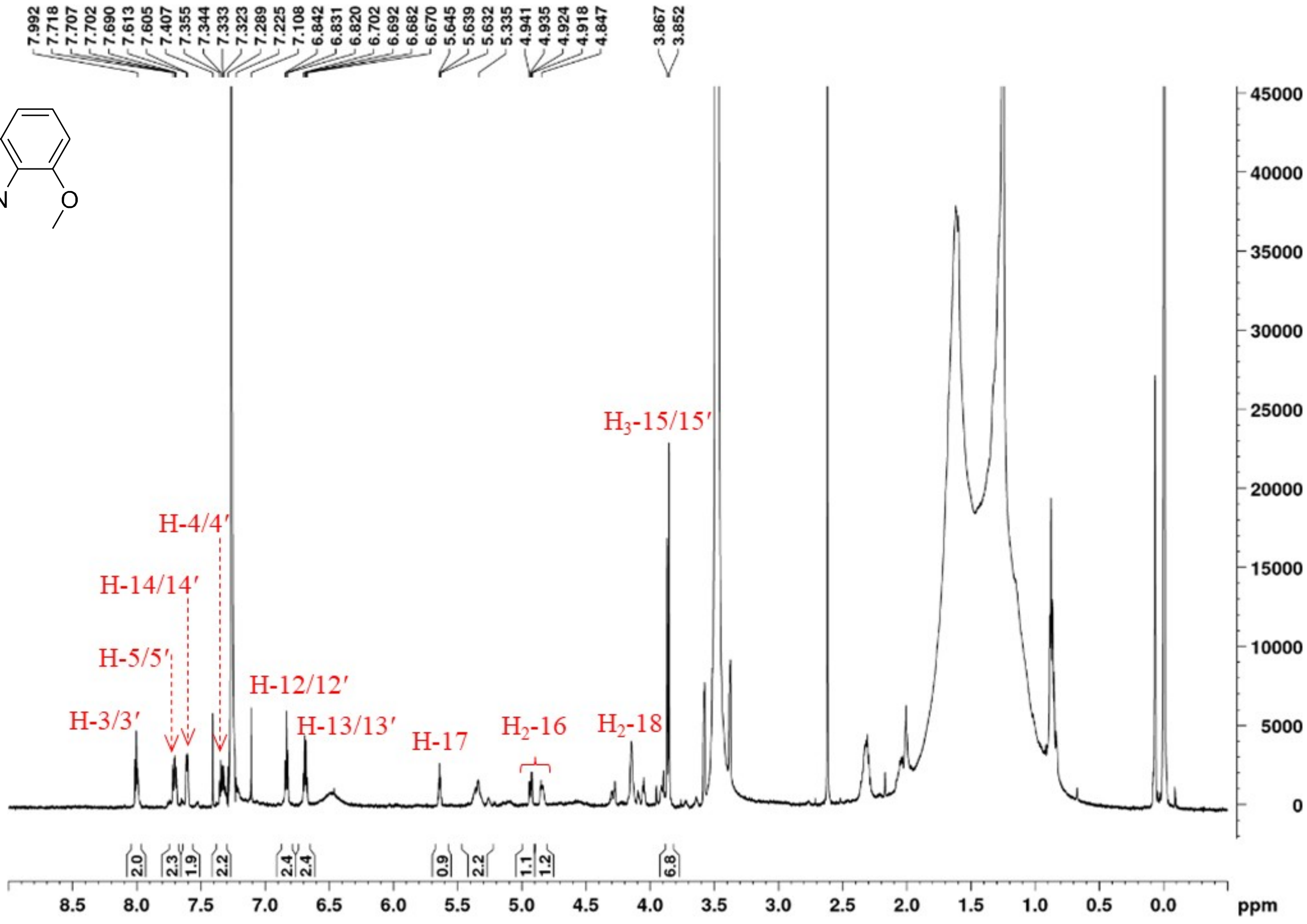

Figure S6. The spectroscopic data of **5**

(C) The^{13}C NMR spectrum of compound **5** in CDCl$_3$ (175 MHz)

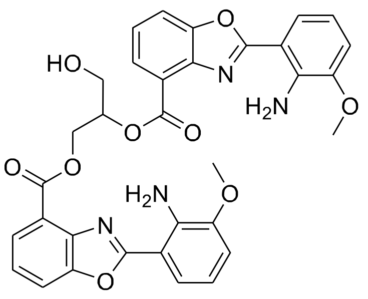

5

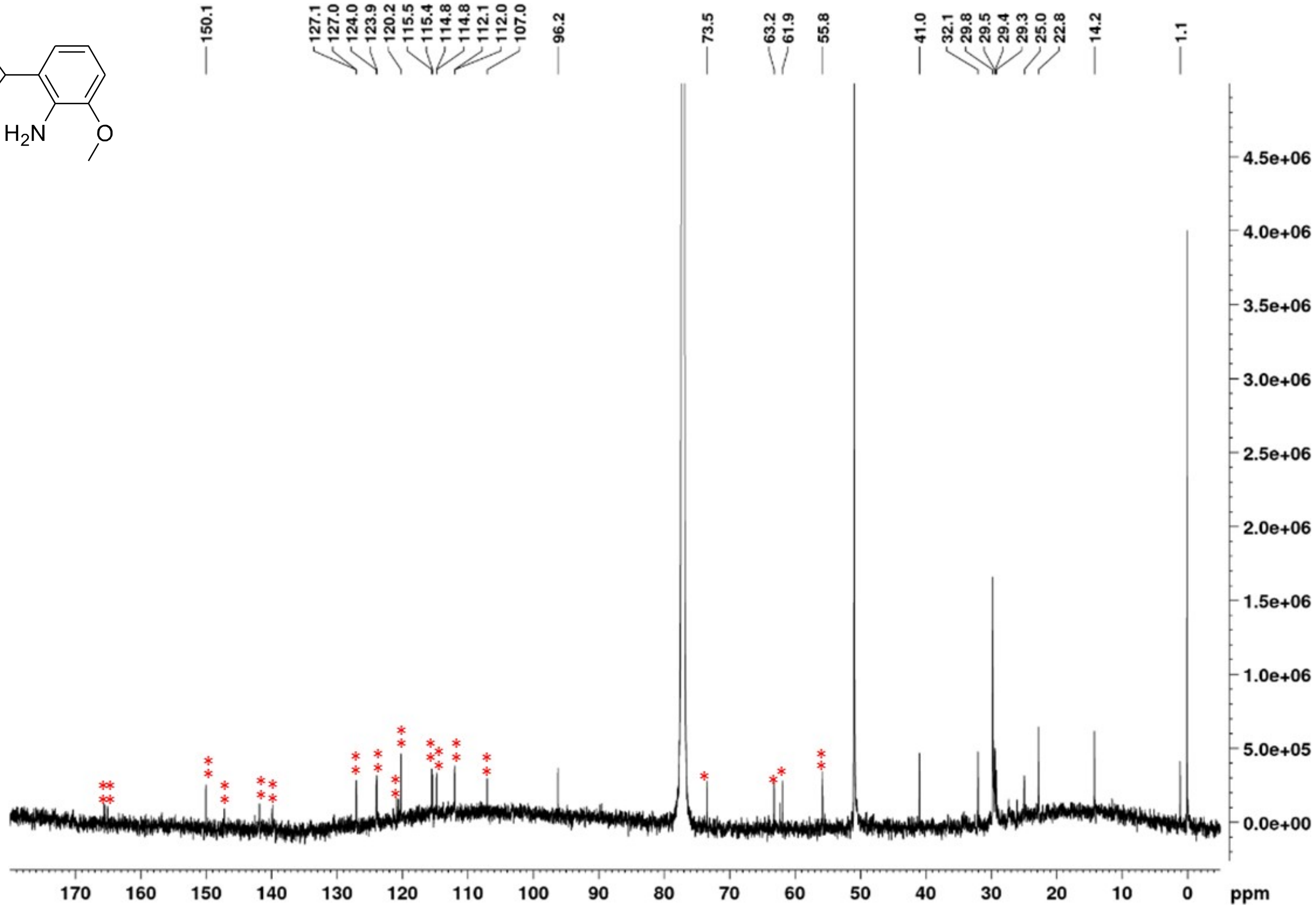

Figure S6. The spectroscopic data of **5**

(D) The DEPT135 spectrum of **5**

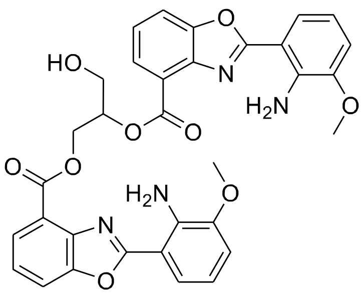

5

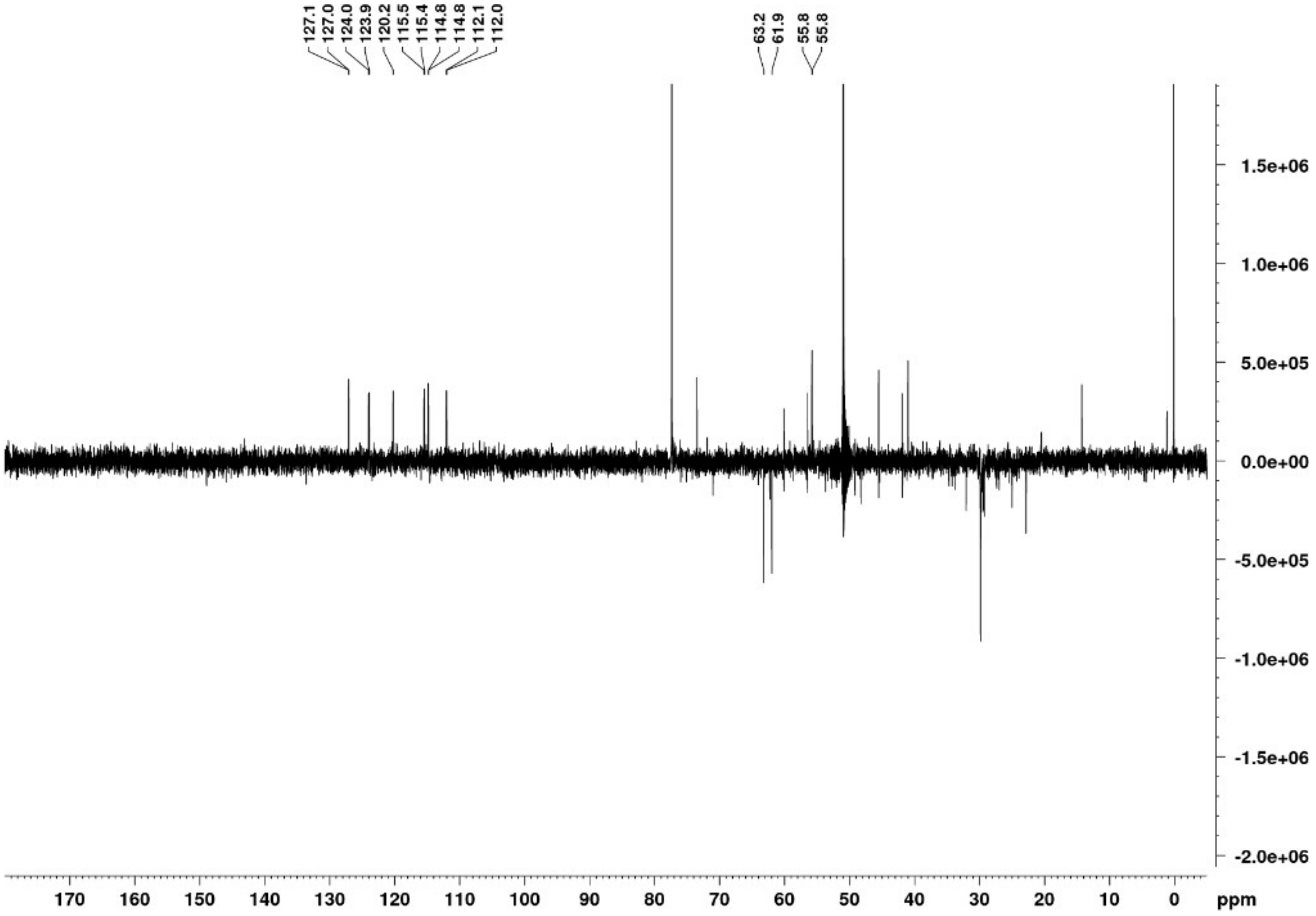

Figure S6. The spectroscopic data of **5**

(E) The HSQC spectrum of compound **5**

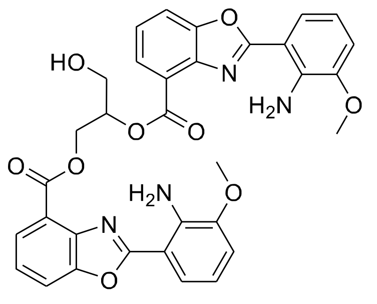

5

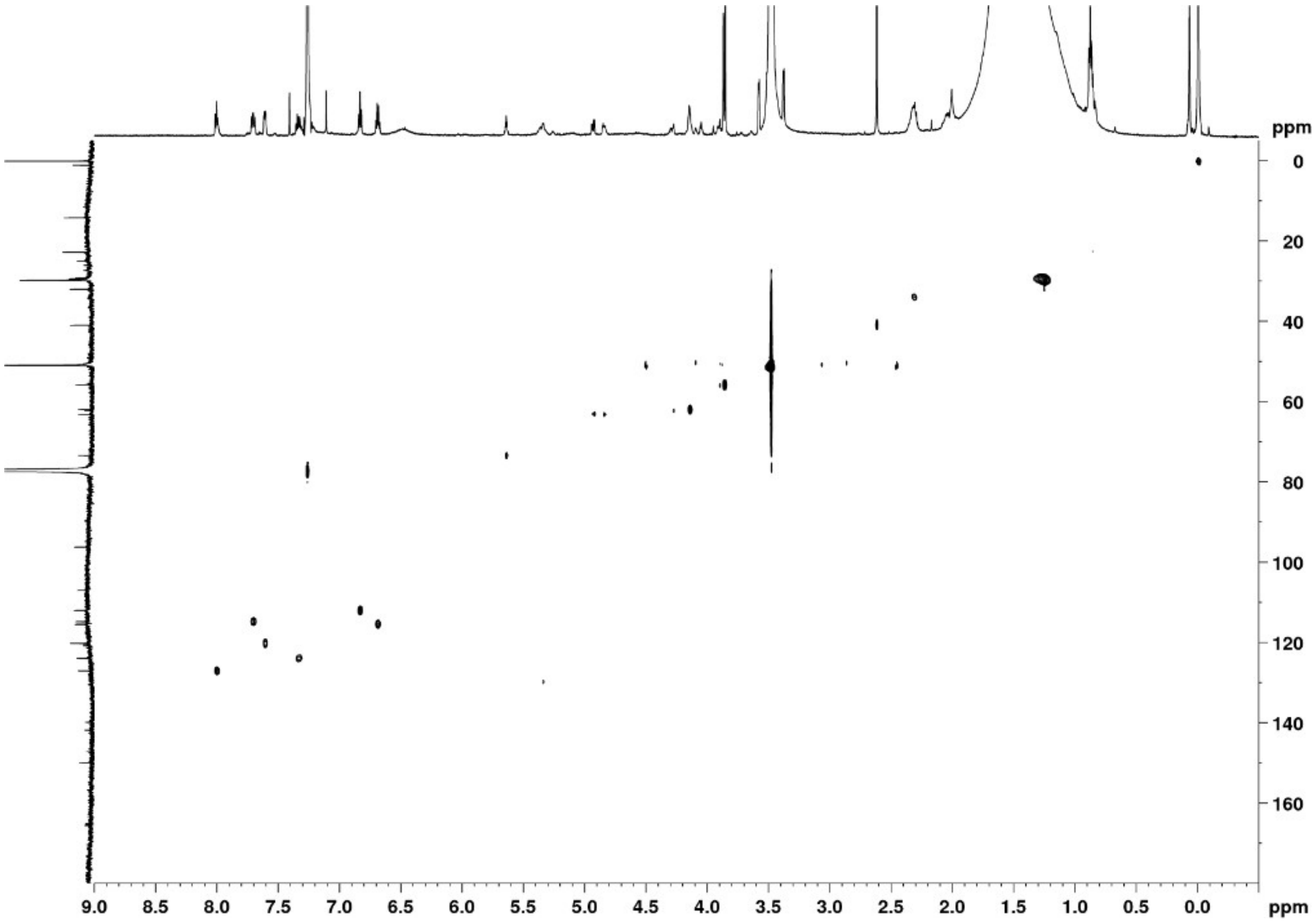

Figure S6. The spectroscopic data of **5**

(F) The HMBC spectrum of **5**

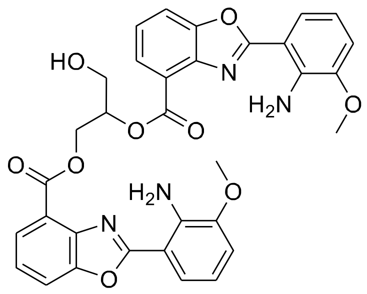

5

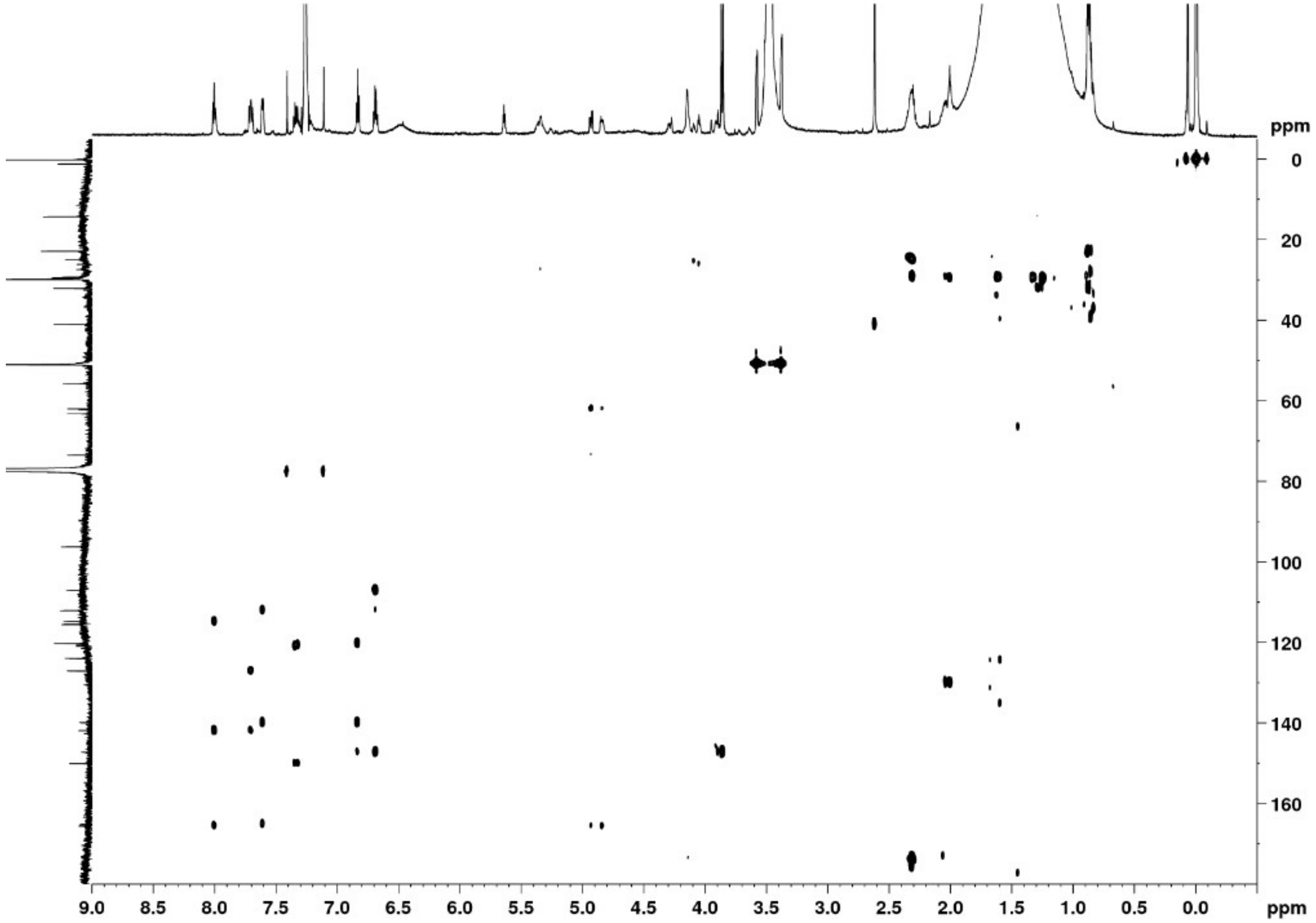

Figure S6. The spectroscopic data of **5**

(G) The ^{1}H-^{1}H COSY spectrum of **5**

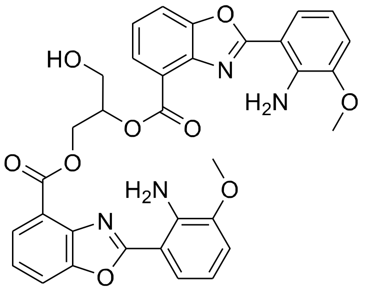

5

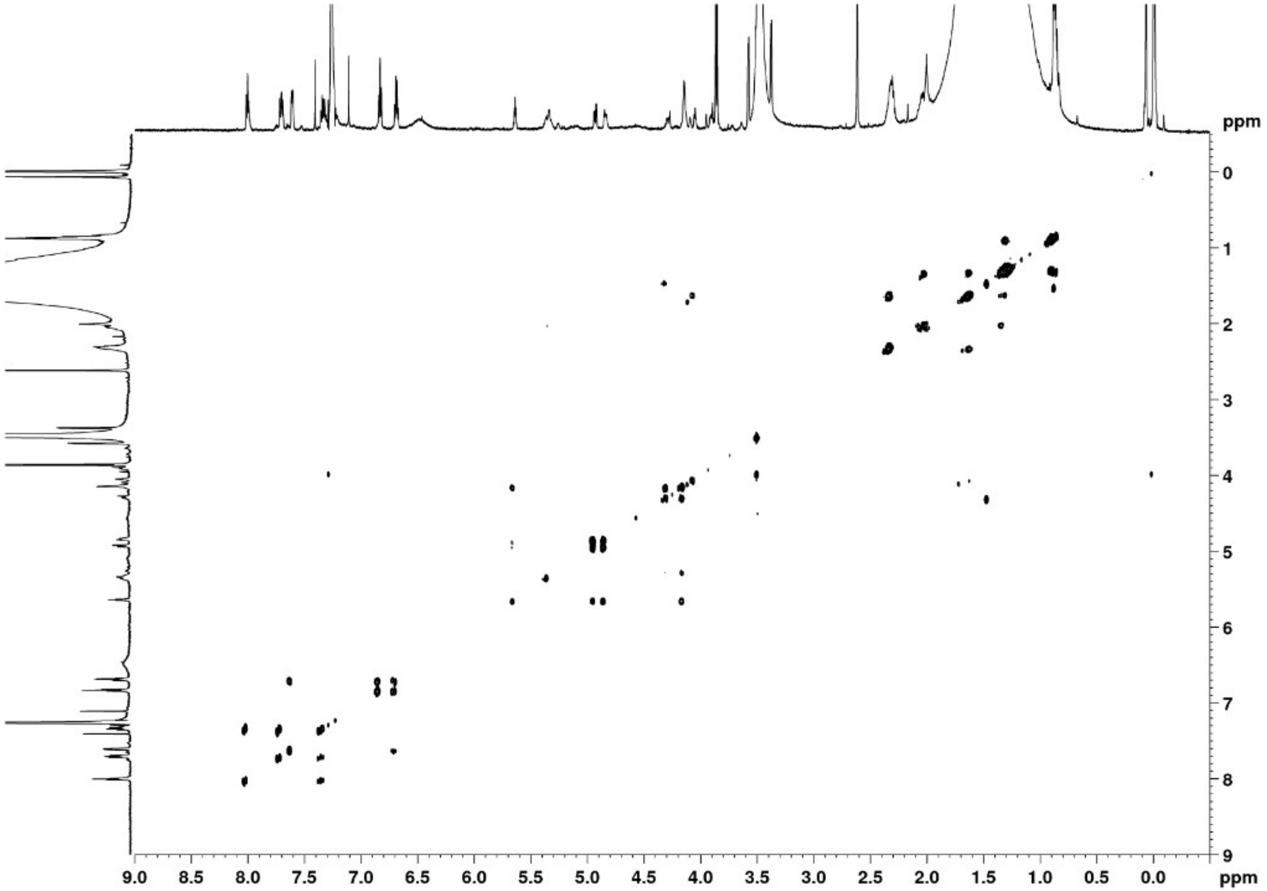

Figure S7. The spectroscopic data of **6**

(A) The HRESIMS (a) UV (b) spectrum of **6**

(a)

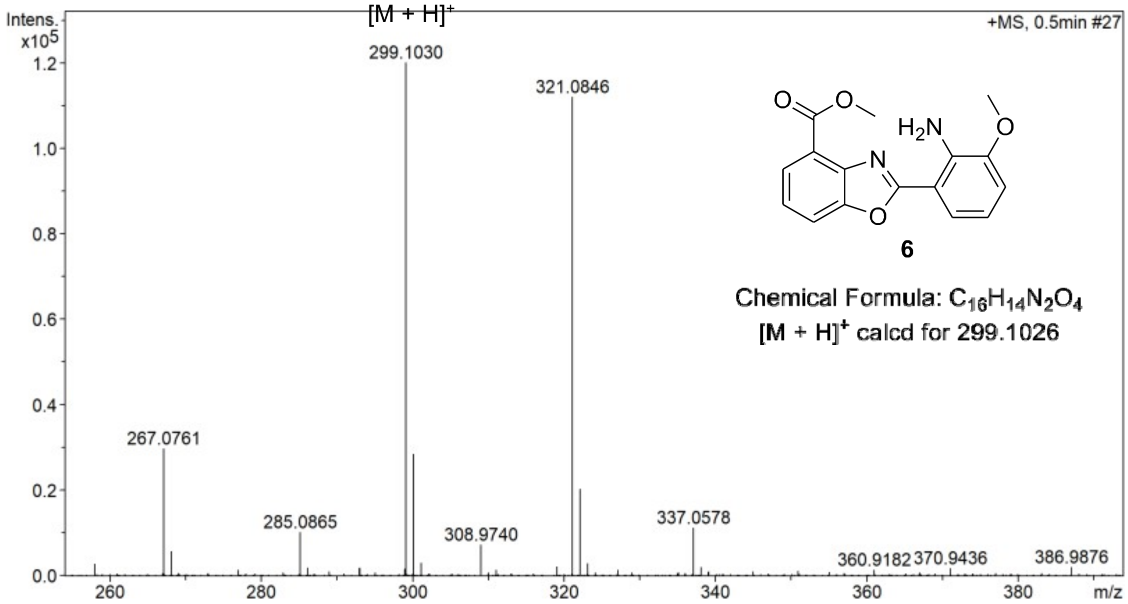

(b)

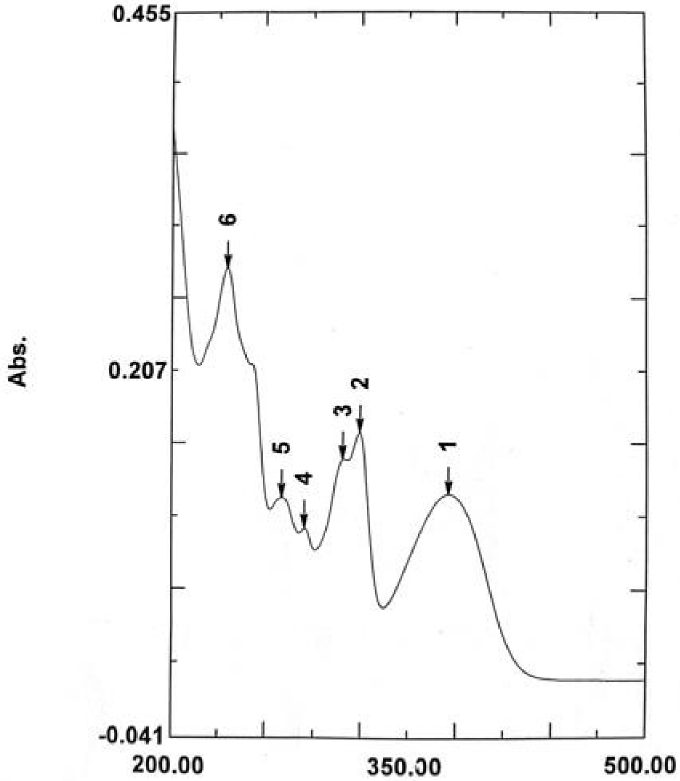

Figure S7. The spectroscopic data of **6**

(B) The ^{1}H-NMR spectrum of **6** (700 MHz for ^{1}H NMR in $CDCl_3$)

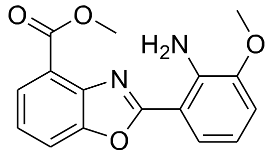

6

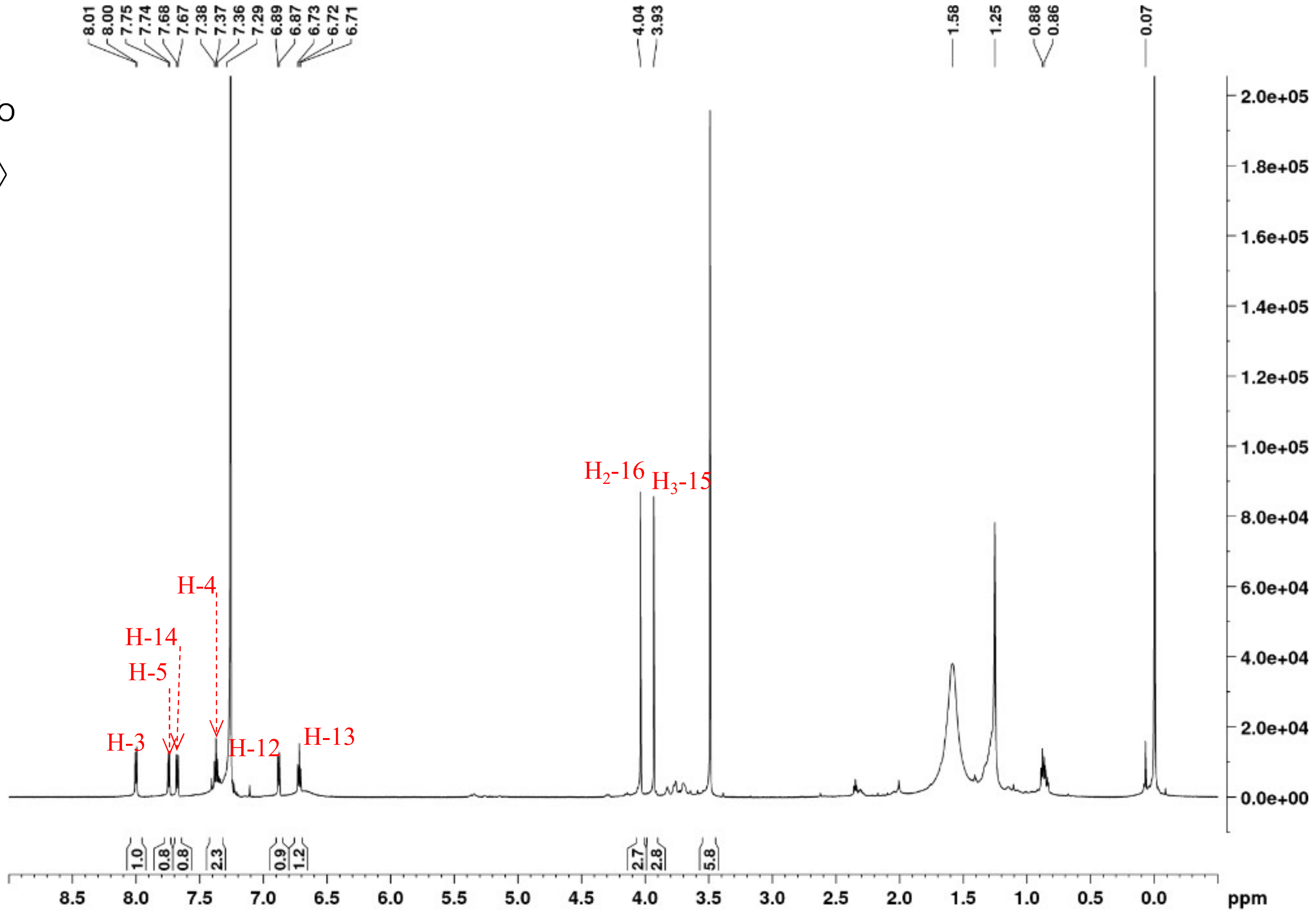

Figure S7. The spectroscopic data of **6**

(C) The ^{13}C-NMR spectrum of compound **6** in $CDCl_3$ (175 MHz)

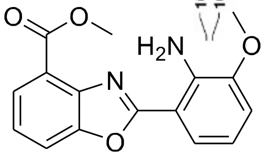

6

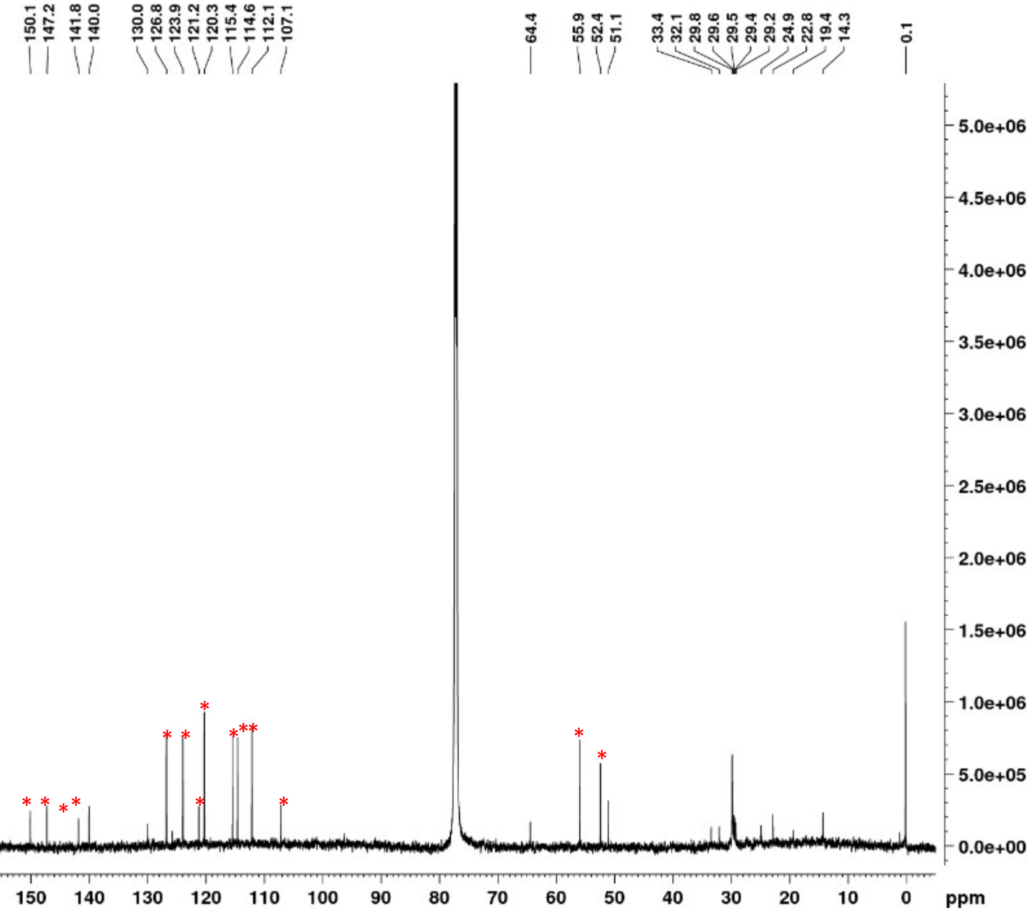

Figure S7. The spectroscopic data of **6**

(D) The DEPT135 spectrum of **6**

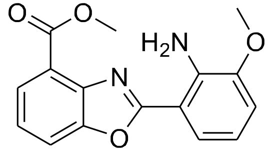

6

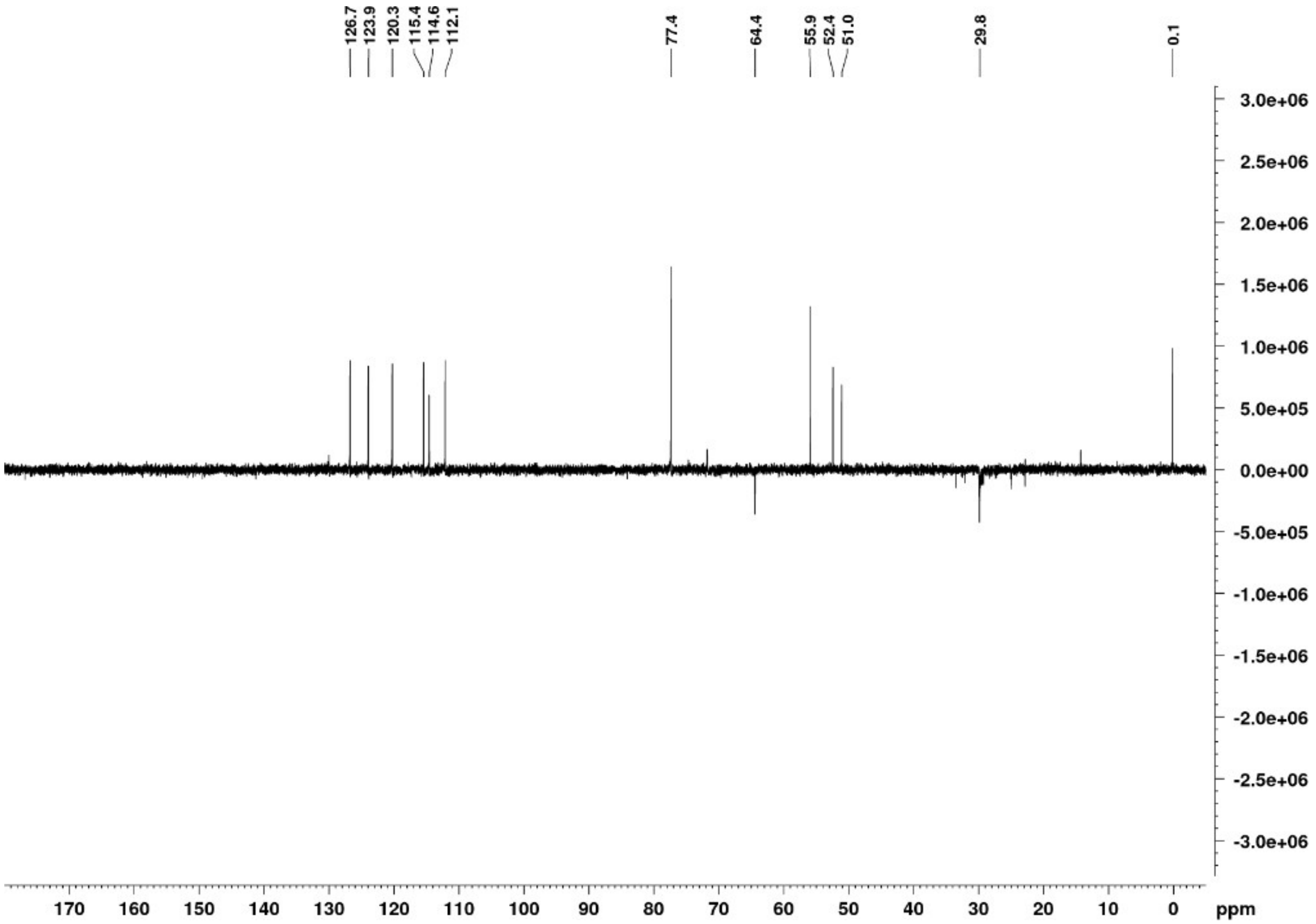

Figure S7. The spectroscopic data of **6**

(E) The HSQC spectrum of compound **6**

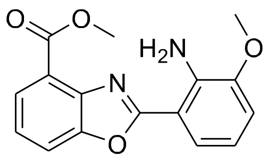

6

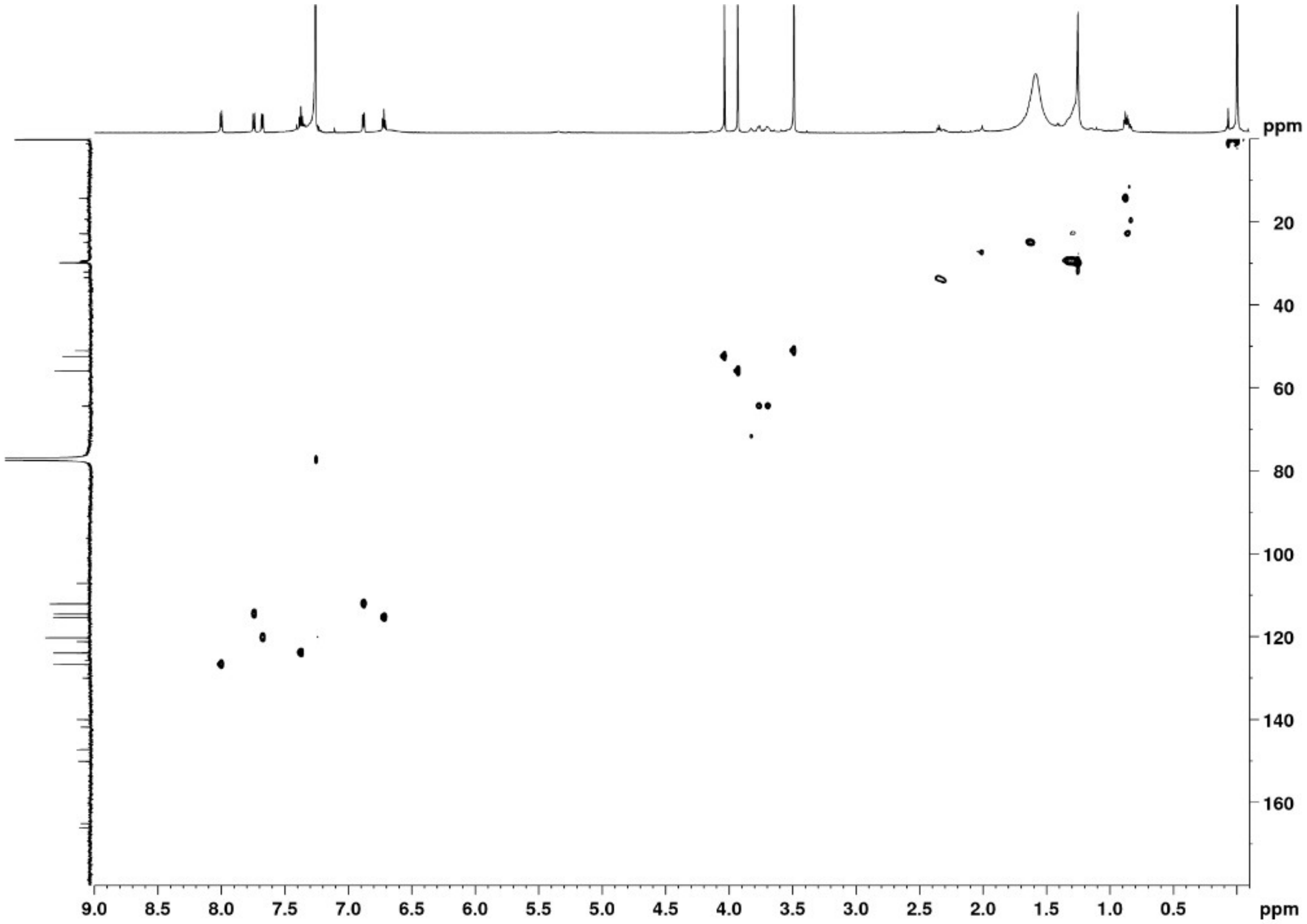

Figure S7. The spectroscopic data of **6**

(F) The HMBC spectrum of **6**

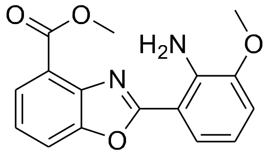

6

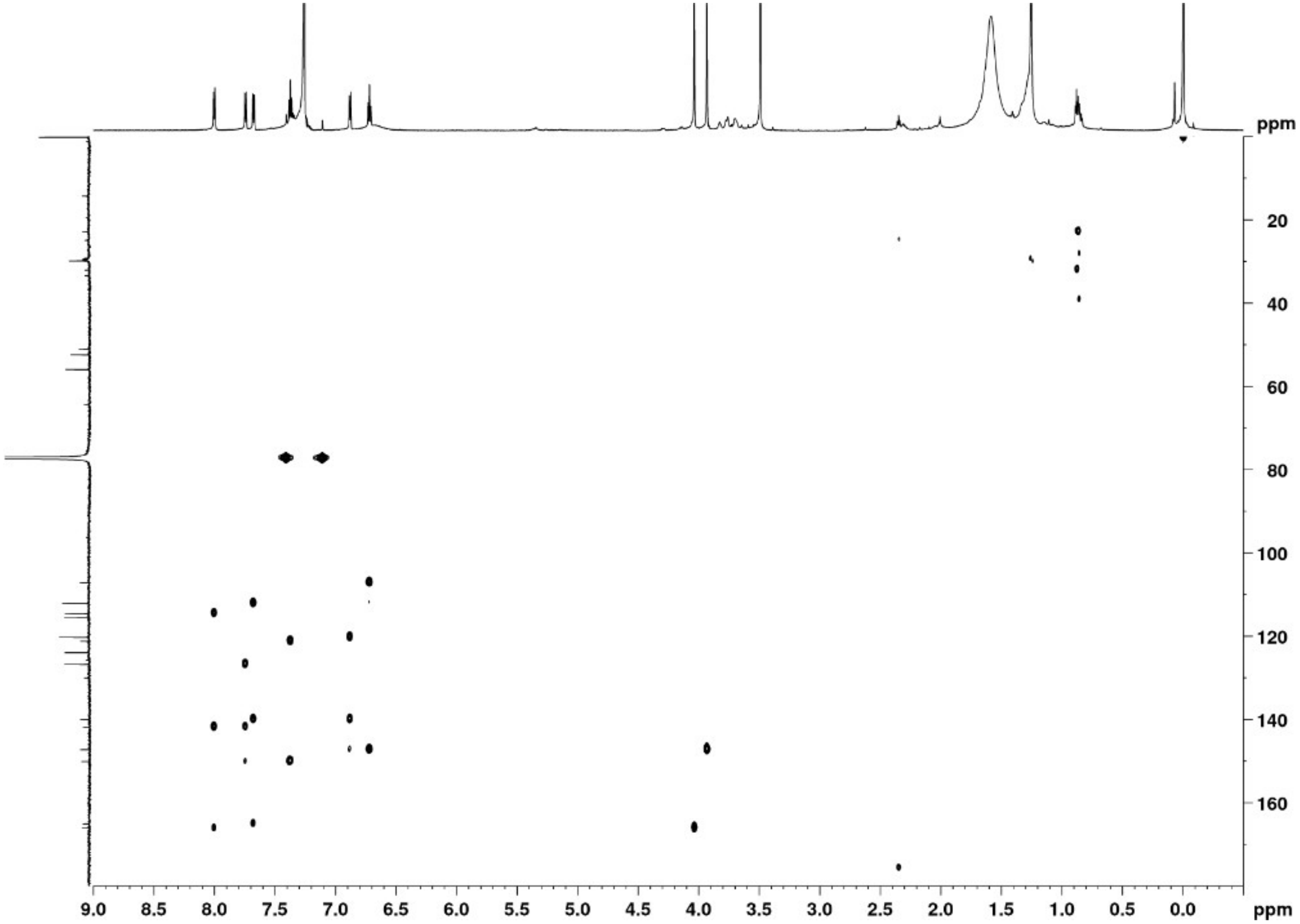

Figure S7. The spectroscopic data of **6**

(G) The ^{1}H-^{1}H COSY spectrum of **6**

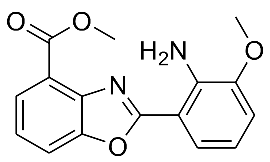

6

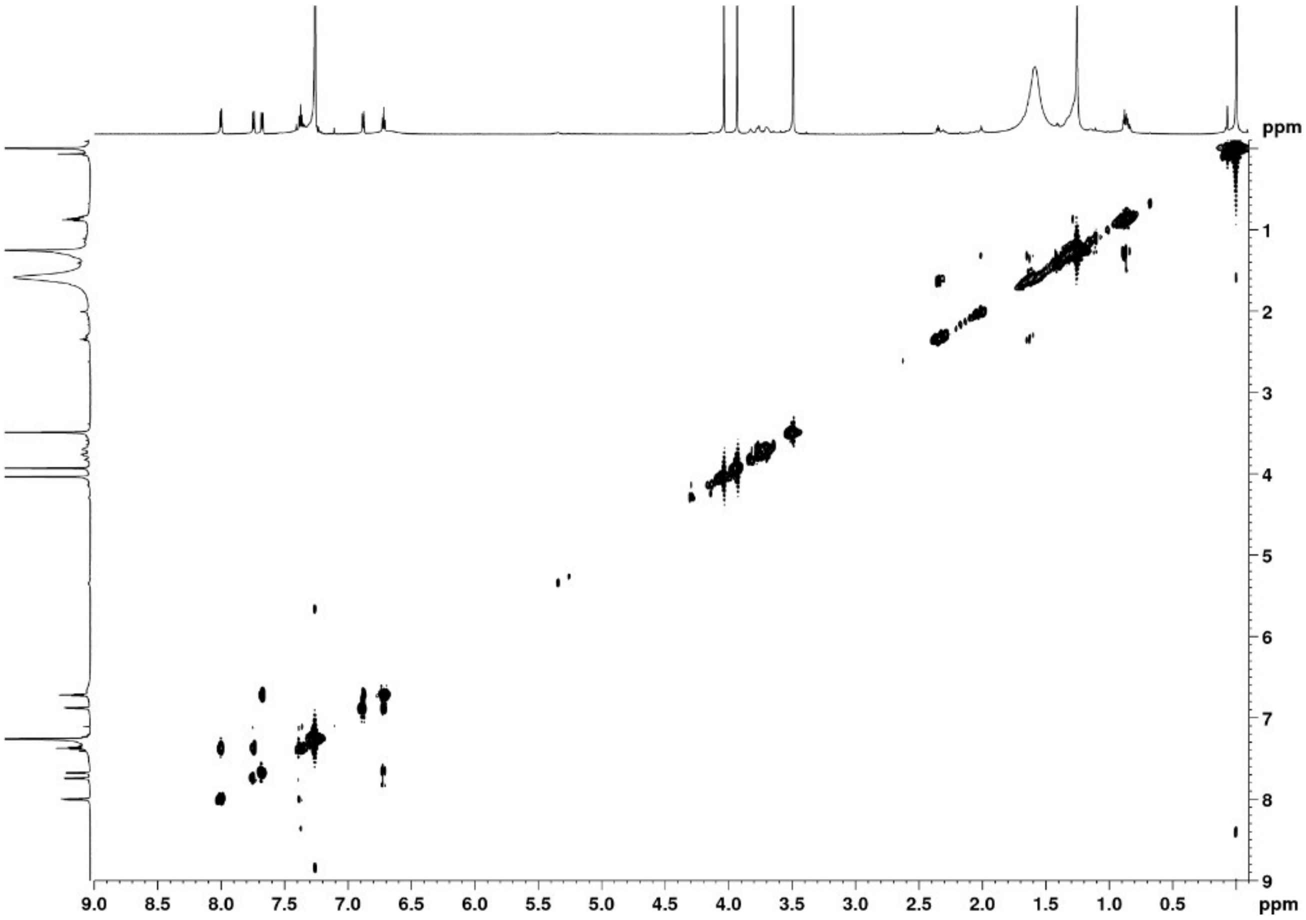

References

1. Flett, F.; Mersinias, V.; Smith, C. P., High efficiency intergeneric conjugal transfer of plasmid DNA from *Escherichia coli* to methyl DNA-restricting *streptomycetes*. *FEMS Microbiol Lett* **1997,** *155* (2), 223-229.
2. Zaburannyi, N.; Rabyk, M.; Ostash, B.; Fedorenko, V.; Luzhetskyy, A., Insights into naturally minimised *Streptomyces albus* J1074 genome. *Bmc Genomics* **2014,** *15*:97.
3. Myronovskyi, M.; Rosenkranzer, B.; Nadmid, S.; Pujic, P.; Normand, P.; Luzhetskyy, A., Generation of a cluster-free *Streptomyces albus* chassis strains for improved heterologous expression of secondary metabolite clusters. *Metab Eng* **2018,** *49,* 316-324.
4. Huang, S.; Li, N.; Zhou, J.; He, J., Construction of a new bacterial artificial chromosome (BAC) vector for cloning of large DNA fragments and heterologous expression in *Streptomyces*. *Wei Sheng Wu Xue Bao* **2012,** *52* (1), 30-37.
